# Exogenous and endogenous dsRNAs perceived by plant Dicer-like 4 protein in the RNAi-depleted cellular context

**DOI:** 10.1186/s11658-023-00469-2

**Published:** 2023-08-07

**Authors:** Paola Leonetti, Arianna Consiglio, Dennis Arendt, Ralph Peter Golbik, Luisa Rubino, Torsten Gursinsky, Sven-Erik Behrens, Vitantonio Pantaleo

**Affiliations:** 1 Department of Biology, Agricultural and Food Sciences, National Research Council, Institute for Sustainable Plant Protection, Bari Unit, Bari, Italy; 2 Department of Biomedical Sciences, National Research Council, Institute for Biomedical Technologies, Bari Unit, Bari, Italy; 3 Institute of Biochemistry and Biotechnology, Section Microbial Biotechnology, Martin Luther University Halle-Wittenberg, Halle Saale, Germany

**Keywords:** RNAi, Yeast, Plant viruses, DCL4, dsRNAs, Antisense transcript, Short RNAs

## Abstract

**Background:**

In plants, RNase III Dicer-like proteins (DCLs) act as sensors of dsRNAs and process them into short 21- to 24-nucleotide (nt) (s)RNAs. Plant DCL4 is involved in the biogenesis of either functional endogenous or exogenous (i.e. viral) short interfering (si)RNAs, thus playing crucial antiviral roles.

**Methods:**

In this study we expressed plant DCL4 in *Saccharomyces cerevisiae*, an RNAi-depleted organism, in which we could highlight the role of dicing as neither Argonautes nor RNA-dependent RNA polymerase is present. We have therefore tested the DCL4 functionality in processing exogenous dsRNA-like substrates, such as a replicase-assisted viral replicon defective-interfering RNA and RNA hairpin substrates, or endogenous antisense transcripts.

**Results:**

DCL4 was shown to be functional in processing dsRNA-like molecules in vitro and in vivo into 21- and 22-nt sRNAs. Conversely, DCL4 did not efficiently process a replicase-assisted viral replicon in vivo, providing evidence that viral RNAs are not accessible to DCL4 in membranes associated in active replication. Worthy of note, in yeast cells expressing DCL4, 21- and 22-nt sRNAs are associated with endogenous loci.

**Conclusions:**

We provide new keys to interpret what was studied so far on antiviral DCL4 in the host system. The results all together confirm the role of sense/antisense RNA-based regulation of gene expression, expanding the sense/antisense atlas of *S. cerevisiae*. The results described herein show that *S. cerevisiae* can provide insights into the functionality of plant dicers and extend the *S. cerevisiae* tool to new biotechnological applications.

**Supplementary Information:**

The online version contains supplementary material available at 10.1186/s11658-023-00469-2.

## Background

Ribonucleic acid (RNA) has multiple roles in cellular functions, from coding genes to non-coding regulatory activities. The latter function is associated with the ability of RNA to form double-stranded (ds) or ds-like secondary structures. DsRNAs accumulate in the host cells in viral infections as a result of viral gene expression and/or viral replication. However, increasing evidence suggests that dsRNAs are not limited to viral origin, but can be produced from endogenous sources, such as retroelements, sense/antisense co-transcriptions [1–3] and, in plants, from the activity of RNA-dependent RNA polymerases.

Plant Dicer-like proteins (DCLs) are RNase III, double-stranded RNA (dsRNA)-specific endonucleases with specialized functions in producing short (s)RNAs of 21- to 24-nucleotides (nt), including micro (mi)RNAs and short interfering (si)RNAs of endogenous or viral origin. In turn, miRNAs and siRNAs guide the effector Argonautes (AGOs)-containing complexes to silence RNA target transcripts. RNA-silencing pathways contribute to viral defense, transposon silencing, heterochromatin formation, and post-transcriptional repression of cellular genes [4, 5].

In the model plant *Arabidopsis thaliana*, DCL1, DCL2, DCL3 and DCL4 have specific roles in the biogenesis of distinct classes of endogenous short (s)RNAs: 21- and 22-nt miRNAs are predominantly generated by DCL1 [6–8], 22-nt siRNAs are produced by DCL2 [9, 10], 24-nt repeat-associated siRNAs from transposons and retro-elements loci, repetitive DNA and reproductive phased siRNAs (phasiRNAs) are produced by DCL3 (and DCL5 in monocots) [11], and 21-nt endogenous trans-acting (ta)siRNAs require the activity of DCL4 [12, 13].

Infections caused by several positive-stranded RNA viruses allow the characterization of DCLs involved in the RNA-silencing-based antiviral immune responses of plants. Viral titers, disease symptoms and the accumulation of sRNAs (i.e. DCL marks) were indeed examined in DCL defective *A. thaliana dcl* mutants. These led to the conclusion that DCL4 and DCL2 act redundantly and one of the two alone is sufficient to perceive dsRNA of viral origin, process it into siRNA of viral origin (vsiRNAs) and initiate the plant RNA silencing-based antiviral defense [14].

The simplest form of RNA silencing, widely known as RNA interference (RNAi), is conserved in diverse eukaryotic species, including the fungal kingdom, but has been lost in the model budding yeast, *Saccharomyces cerevisiae* [15]. *S. cerevisiae* is among the few eukaryotes that do not express the known components of the RNAi machinery, which normally occurs in the cytoplasm [16]. Accordingly, inducible RNA degradation is not initiated by RNAi in *S. cerevisiae*, and *rnt1* is the only gene encoding a nuclear endonuclease with a unique recognizable RNase III motif [17, 18]. Ribonuclease three 1 (RNT1) is essential for ribosome synthesis as it involves the processing of ribosomal RNA (rRNA) and has a minor impact on mRNA processing in the nucleus [18].

The yeast system has proved extremely suitable for fundamental studies in virology. Indeed, stably expressed replicase proteins of plant and animal viruses can reconstitute active membrane-associated replication complexes. When replication-competent genomic or sub-genomic RNAs [19, 20] or subviral defective-interfering (DI)-RNAs [21, 22] are introduced into yeast cells they are recruited to the replication complexes and actively replicate. Furthermore, it is possible to reconstitute the RNAi process in *S. cerevisiae* by introducing Dicer and AGO proteins from *Homo sapiens* or *S. castellii*; i.e., the reconstituted silencing pathway was shown to knock down a reporter RNA and endogenous retrotransposons, respectively [16, 23].

In plants, many crucial steps of antiviral RNA silencing have not yet been fully studied. For instance, redundancy and hierarchical action of DCLs in *A. thaliana* and DCL duplications and subfunctionalization [24] all together hamper studies aimed at characterizing individual functions of plant DCLs in antiviral defense. Moreover, localization studies using green fluorescent protein (GFP) fusion proteins indicate that all four *Arabidopsis* Dicers localize to the nucleus where they most likely generate endogenous sRNAs [12, 25, 26], whereas RNA plant viruses replicate entirely in the cytoplasm associated with membranes; how these nuclear enzymes use cytoplasmic substrates to produce vsiRNAs is still unknown.

Given that *S. cerevisiae* lacks the RNAi pathway and supports viral RNA replication, it makes the budding yeast an ideal heterologous system to shed light on the obscure steps of the plant antiviral RNAi pathway. In the present study we have stably expressed the plant DCL4 in *S. cerevisiae* and investigated some aspects of its functionality. For instance, cell extracts from DCL4-expressing *S. cerevisiae* are able to process viral dsRNAs or dsRNA-like molecules into sRNAs in vitro. In vivo, DCL4 processes a transcribed hairpin RNA into 21-nt and, secondarily, into 22-nt sRNAs. Unexpectedly, DCL4 is not able to process a viral replicase-assisted DI-RNA replicon. These findings have biological implications, suggesting the importance of additional factors or events in the initiation of the antiviral immune response in plants. It is worth mentioning that the conspicuous presence of sense and antisense sRNAs associated with specific cellular loci suggests that DCL4 is able to recognize cellular sense/antisense co-transcribed RNAs that form perfect dsRNAs. The effects of DCL4 on endogenous RNAs of *S. cerevisiae* allowed the validation of decoded but not yet annotated sense/antisense loci and the discovery of at least three previously unknown loci.

## Materials and methods

### Yeast strain and expression plasmids

*S. cerevisiae* strain YPH499 (MATa *ura3*-*52 lys2*-*801 ade2*-*101 trp1*-*63 his3*-*200 leu2*-*1*) was transformed with plasmid DNAs by using the lithium acetate-polyethylene glycol method [21]. Following transformation, cells were grown and maintained on appropriate synthetic selective medium (SM) plates containing 2% dextrose [27]. Relevant amino acids were omitted to maintain the selection for any plasmid used. Cymbidium ringspot virus replicase proteins p33/p92 were expressed under control of the alcohol dehydrogenase gene 1 (ADH1) promoter and the terminator of vector denoted as pA, containing the 2 μm origin of replication and the *HIS* as a selectable marker [28]. Cymbidium ringspot virus DI-3 [29] was used as a replication template. It was expressed by the galactose-inducible (GAL1) promoter and the ADH1 terminator into the low-copy-number centromeric plasmid vector pBMI3S containing tryptophan as a selectable marker [28].

For the generation of the DCL4 expression vector, total RNA was extracted from 100 mg leaf tissues of *Nicotiana benthamiana* with TRIzol (Invitrogen) following the manufacturer’s instructions. Complementary (c)DNA was then obtained using revert Aid H Minus RT (Thermo scientific) and Oligo(dT) primer using the manufacturer’s instructions. DCL4 ORF was PCR-amplified using cDNA as template and subcloned into pSPLF2, a modified PSP64 Poly(A) vector (Promega). The DCL4 (BankIt2712565, NbDCL4 accession n. OR12626) was then PCR-amplified with the forward oligo 5′-ACGCGTCGACATGACGCTGGTGGTAGCGGTGGTAGC-3′ flanked by the restriction site of *SalI* (underlined) and reverse oligo 5′-AGCGGCCGCGGTCAGTTATCGAACATGTATCC-3′ flanked by the restriction site of *NotI* (underlined). The PCR fragment was *SalI*/*NotI* digested and cloned into *XhoI/NotI* digested CEN6/ARS14 pSAL1 containing *LEU2* as a selectable marker [30]. In order to place the hemagglutinin (HA) epitope at the 5′-end of the DCL4 ORF, a two steps site-specific mutagenesis was then applied to the plasmid pSAL1-DCL4 using the oligos HAstep1for 5′-CCATGGACTACAAGGACGACGACGACAAGCGGTGGTAGCGGTGGTTCCGAAGGCGGCTACTTTG-3′ and HAstep1rev 5′-CAAAGTAGCCGCCTTCGGAACCACCGCTACCACCGCTTGTCGTCGTCGTCCTTGTAGTCCATGG-3′ for the step 1 and FinalHAfor 5′-CGACTCTAGAATTACCATGTACCCATACGATGTTCCAGATTACGCTGGTGGTAGCGGTGGTAGCGG-3′, FinalHArev 5′-CCGCTACCACCGCTACCACCAGCGTAATCTGGAACATCGTATGGGTACATGGTAATTCTAGAGTCG-3′, for the step 2. Site-directed mutagenesis on plasmids was performed with the QuikChange site-directed mutagenesis kit (Stratagene) to generate the plasmid pSAL-HADCL4. All mutations were confirmed by DNA sequencing. Plasmid pRS403-PGAL1-strongSC_GFP, which expresses the GFP hairpin named in the text Strong Silencing Construct (SSC) RNA, was previously described by Drinnemberg et al. [16].

## RNA structure analysis

Positional sequence entropy plot, secondary structure and color map of base-pair probabilities of the hairpin from the plasmid pRS403-PGAL1-strongSC_GFP transcript was calculated using RNAFold webserver at ViennaRNA pakage [31].

### Growth conditions

Yeast cultures were grown at 25°C if not otherwise indicated. For induction of the CymRSV DI-3 (see above) [29], cells were first subcultured into SM containing 2% dextrose then into 3% glycerol–0.1% dextrose and successively GAL1 promoter was induced in SM containing 3% glycerol–2% galactose. DCL4 under the copper chelation protein 1 (CUP1) promoter was induced by adding CuSO_4_ at a final concentration of 0.25 mM.

### Protein extraction and RNA analysis

A volume of yeast cultures containing 2 units of optical density at 600 nm and harboring selected plasmids as indicated grown until the mid-logarithmic-phase (optical density at 600 nm, 0.6 to 0.8), were harvested by centrifugation and processed for total protein extraction using the previously described protocol [32] or RNA analysis as previously described [21]. HA-DCL4 was detected by western blot analysis with mouse anti-HAtag mAbs (probe sc-7392, Santa Cruz Biotechnology). 

Total RNA was prepared by the hot-phenol method and resuspended in RNase-free water, and 2 μg of RNAs were used for sRNA and transcriptome analyses. Libraries of sRNAs were produced using a small RNA seq Kit (Bioo Scientific) and sequenced with standard sequencing oligos on the Illumina HiSeq 2500 platform. Libraries for RNAseq were prepared using TruSeq Stranded Total RNA Prep kit (Illumina) and sequenced by Illumina NovaSeq 6000. 

### qRT-PCR

For quantification of gene expression of yeast grown overnight at 25°C, with 0 μM and 250 μM [Cu^2+^], cDNAs were synthesized from 1 μg of total RNA extracted using the QuantiTect Reverse Transcription Kit (Qiagen) with the gene-specific primers: *GFP*, 5′-TGGAAGCGTTCAACTAGCAGA; *ACT1*, 5′-TCATGGTCGGTATGGGTCAA, following the manufacturer’s instructions. qRT-PCR reactions were carried out by means of a StepOnePlus real-time system (Applied Biosystems) and assembled in reaction with 1.5 μl cDNA, 10 μl SYBR^®^ Select Master Mix (Applied Biosystem), 0.2 μl each of 100 mM of forward and reverse primer, and RNAse free water to 20 μl (total volume). Thermocycling was carried out with one cycle at 95°C for 10 min, followed by 40 cycles of 95°C for 45 s and 58°C for 1 min and 72°C for 45 s. The dissociation curve of the final products was checked to ascertain the presence of a single amplification product. qRT-PCR was performed on triplicate samples of each cDNA using the following primers: Hp*GFP*, 5′-TGGAAGCGTTCAACTAGCAGA and 5′-CTAATTGGTAAAGATAGAGGAATTCGT; *ACT1*, 5′-TCATGGTCGGTATGGGTCAA and 5′-CCATATCGTCCCAGTTGGTGA, the *S. cerevisiae* Actin gene was selected as reference gene (GenBank: MK879550.1). Gene expression was calculated using the Delta–Delta cycle threshold method [33, 34].

### DCL4 immunoprecipitation and isolation for in vitro assays

**Fig. 1:**
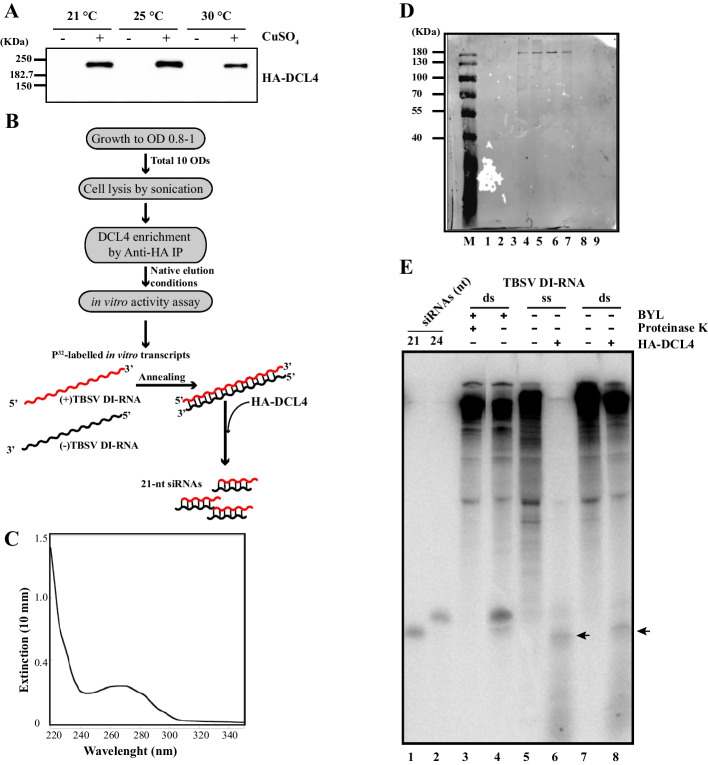
Expression in *S. cerevisiae* and in vitro functionality of recombinant DCL4. **A** Western blot analysis of whole cell lysates, separated in an 8% polyacrylamide gel, showing HA-epitope-tagged DCL4 expression in *S. cerevisiae* after 24 h growth at 21°C, 25°C and 30°C, respectively. **B** Flow chart used for DCL4 enrichment and in vitro functionality. **C** Spectrophotometric curve of the DCL4-enriched yeast fraction (DEYF). **D** Western blot analysis of DEYF: PageRuler Prestained protein Ladder (M), total yeast lysate (lanes 1–3), eluted fractions from anti-HA agarose-conjugated columns (lanes 4–6), DEYF fraction > 50 kDa (lane 7), flow-through of the concentration step < 50 kDa (lane 8), HA peptide in TBS-Tween (lane 9). **E** In vitro functionality of DEYF in processing single-stranded and double-stranded defective-interfering (DI) RNA transcripts of Tomato bushy stunt virus. Arrows highlight the sRNAs

*Saccharomyces cerevisiae* strain YPH499 carrying pSAL-HADCL4 plasmid was sub-cultured in 10 mL of -LEU minimal media containing 2% dextrose at 30°C. The subculture was scaled up to two liters of the same medium containing 0.25 mM copper sulfate and grew O/N at 25°C until reaching the optical density at 600 nm of 0.8. Yeast cells were pelleted and frozen in dry ice upon storing at − 80°C. Frozen cells were resuspended in 5 mL TBS-Tween (25 mM Tris–HCl pH 7.2, 150 mM NaCl with 1 tablet (per 25 mL) of Complete EDTA-free protease inhibitors (Roche), with Tween^®^-20 (Merk) to a final concentration of 0.05%) in 15 mL Falcon tubes and then sonicated (Bandeline Sonoplus UW2070, Bandelin Electronic) in ice using output amplitude of 70%, 30 s per cycle for a total of 5 times per 2.5 min. The yeast lysate was splitted in four 2 mL eppendorf tubes and centrifuged at 16,000*g* for ten minutes at 4°C. The supernatant was transferred in a new 15 mL Falcon tube. The lysate was diluted 1:10 with TBS to a final concentration of Dithiothreitol (DTT) of 1 mM in a 50 mL Falcon tube and kept in ice. 0.4 mL of agarose-antiHA gel (Thermo Fisher), were washed twice with the same volume of TBS and added to 50 mL falcon tubes containing the yeast lysate. The yeast lysate was incubated with the agarose-antiHA gel O/N on the wheel at 4°C. The Falcon tubes have been centrifuged at 12,000 *g* for one minute and the pellet was washed three times with 1 mL ice-cold TBS-Tween (w/o DTT). The pellet was resuspended by gentle pipetting with wide-bore tips in 0.5 mL of TBS containing 1 mg/mL of HA peptide (Thermo Scientific) and incubated at 25°C with a slow shaking for 15 min. After centrifuging at RT for ten seconds at 12,000 *g*, the procedure was repeated for additional three times, thus obtaining three aliquots of supernatant (see Fig. 1E, lanes 5–7) which contained 90% of the eluted HADCL4. One Corning SPINX tube (UF6 cat n.431483) was then equilibrated with two mL of TBS, and the three HADCL4-containing supernatants were subsequently added to the Corning tube and centrifuged at 10,000 *g* for 5′ at 10°C. The eluted DCL4-enriched yeast fraction (DEYF) of 0.1 mL was transferred to a new eppendorf tube and kept on ice until used for the in vitro assay.

## In vitro assays

*Nicotiana tabacum* BY2 cells were cultured at 23°C in Murashige–Skoog liquid medium. Evacuolated BY2 protoplasts were obtained by percoll gradient centrifugation and cytoplasmic extract (BYL) prepared as described earlier [35]. Single- or double-stranded ^32^P-labeled in vitro transcribed RNA (1.5 pmol) [36] was incubated in a 20-µl reaction containing 50% (v/v) BYL under translation conditions [36] or 5-µl DEYF. The RNA was isolated from the reactions as described previously and resolved on 15% denaturing Tris–borate polyacrylamide gels. ^32^P-labeled products were visualized by phosphor-imaging.

### Bioinformatic and statistical analysis of sequenced data

FASTQ quality was checked using FastQC [37]. RNA-Seq data were mapped with STAR [38] on *S. cerevisiae* S288C (genome available at NCBI, RefSeq assembly n.R64); multireads were evaluated with RSEM [39] and MultiDEA [40] tools; differential expression analysis was performed with DESeq2 [41], which uses Benjamini–Hochberg for p-value correction. The changes in expression were considered statistically significant if the adjusted p-value was < 0.05, the mean read count was > 50 and the absolute fold change was > 1 (absolute log2 FC > 0.585). The adapters of sRNA-Seq reads were removed with Cutapt [42]. sRNA-Seq data were mapped with Bowtie [43] on the SSC RNA sequence and on NCBI reference searching for perfect alignments (no mismatch allowed); Fisher’s Exact test was applied to study the expression of sRNA originating from the SSC RNA, and in particular to test their nucleotide subsequence occurrence in expression peaks and to compute the distance from the hairpin loop from were sRNA expression significantly decrease; BASH, Python and R custom scripts were used for additional data manipulation, statistical analyses and custom plots; read alignments were displayed through the UCSC Genome Browser [44].

### TAS2-derived sRNAs

4 replicas of sRNA datasets from *A. thaliana col0* wild type, *dcl2-4*, *dcl4-1* single mutants and *dcl4dcl2* double mutants [45] are from https://www.ncbi.nlm.nih.gov/geo/ under accession number GSE136164. Row data underwent the elaboration used for SSC RNA-derived sRNAs and were aligned to TAS2 cDNA (locus AT2G39681).

## Results

### Plant DCL4 expression induction in budding yeast and in vitro activity

*Nicotiana benthamiana* is widely considered a model plant in plant virology, the recognized host for study tombusviruses [22, 29, 46]. Human influenza hemagglutinin-tagged (HA)-DCL4 was expressed via an episomal plasmid under the control of the copper chelation protein 1** (**CUP1) inducible promoter (see “Materials and methods” section) in *S. cerevisiae* grown at 21°C, 25°C and 30°C (Fig. 1A). Considering that the expression and functionality of plant antiviral DCLs is temperature-dependent and particularly active at temperatures of 25–27°C [47], we choose 25°C as the standard temperature for further investigations.

Prior to functional analyses, to concentrate the soluble protein, the cell lysates were subjected to anti-HA small affinity purification (see flowchart in Fig. 1B and details in “Materials and methods” section). The fraction eluted from the chromatographic column contained a DCL4-enriched yeast fraction (DEYF) with a spectrophotometric curve denoting protein enrichment, although not absolute purity (Fig. 1C), and Western blot analysis confirmed the enrichment of DCL4 in the DEYF (lanes 5–7, Fig. 1D).

To test whether the recovered HA-DCL4 was functional, we incubated DEYF at 25°C with typical substrates of plant DCLs, either a highly structured single-stranded (ss) DI-RNA [46] or a perfect dsRNA. For this purpose, we applied radioactively labeled in vitro transcribed sense and antisense transcripts of a DI-RNA of the tomato bushy stunt virus as depicted in Fig. 1B and in "Materials and methods". As a positive control, we performed the same experiment with the cytoplasmic extract of evacuolated *Nicotiana tabacum* BY-2 protoplasts (BYL); BYL was previously shown to contain endogenous DCL activities with a predominant action of DCL3 [48]. Interestingly, DEYFs were capable of processing the offered dsRNA-like substrates or dsRNA into sRNAs (arrows in Fig. 1E). However, unlike BYL, which produced mainly DCL3-dependent 24-nt sRNAs, processing in DEYF generated mainly sRNAs of 21 nt.

### In vivo, DCL4 efficiently processes hairpin constructs

Subsequently, we tested whether DCL4 was capable of processing RNA substrates in vivo under cell growth conditions. For this purpose, we co-expressed DCL4 with an RNA molecule as a substrate, which has been earlier determined as a ‘strong silencing construct’ (SSC) of the mRNA reporter of GFP as a means of inducing processing by *S. castellii* DCR1 into 23-nt sRNAs [16].

**Fig. 2:**
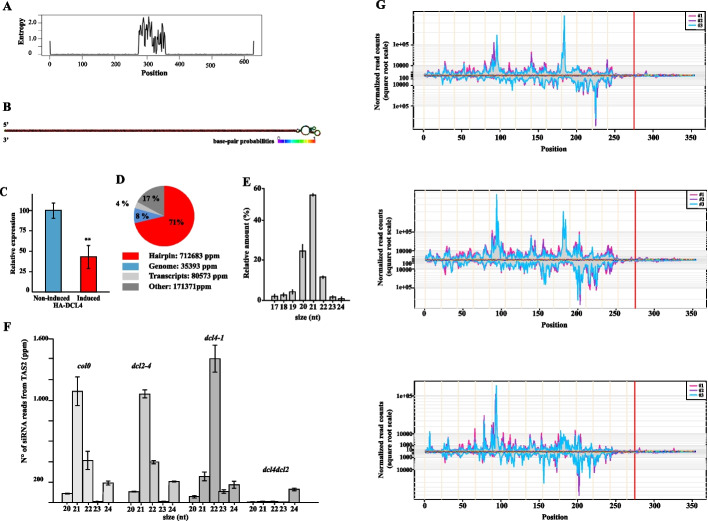
In vivo functionality of recombinant DCL4 in *S. cerevisiae* on an inducible hairpin transcript. **A** Positional sequence entropy plot of the hairpin transcript, 275-nt long constituted by gfp inverted repeats separated by the 79-nt *RAD9* intron (with max entropy values). **B** Secondary structure and color map of base-pair probabilities of the hairpin transcript. **C** Relative expression level by qRT-PCR of the galactose-induced hairpin transcript in the absence and presence of copper-induced DCL4. Data are shown as the mean value SD from three independent assays using Student’s t-test (*P < 0.05; **P < 0.01). **D** Relative abundance of sRNAs from the SSC hairpin transcript, *S. cerevisiae* reference genome and transcripts. The relative abundance is also expressed in average part per million (ppm) referred to three replicates of sRNA sequencing. **E** Size distribution profile of 17-to-24 nt sRNAs from SSC hairpin transcript. **F** Size distribution profile of 20-to-24-nt sRNAs from TAS2 transcripts in *A. thaliana * Col-0 WT, and in mutant lines containing *dcl2-4*, *dcl4-1* single mutants alleles and *dcl4dcl2* double mutants alleles. **G** Distribution of 20-, 21- and 22-nt sRNAs (upper, middle and lower panels, respectively) along the hairpin transcript. Pink, violet and cyan lines refer to three independent replicates of short RNA sequencing. 5′-end positions of normalized sRNA reads were plotted using a square root scale. The red vertical line indicates the border of rad9 intronic loop

The SSC consists of two complementary regions corresponding to a portion of the GFP mRNA separated by the *RAD9* intron [16]. Upon galactose-induction, SSC produced a 629-nt RNA transcript forming a hairpin conformation with a high degree of nucleotide pairing and low entropy level (Fig. 2A and B). Accordingly to previous indications [21], after GAL1 induction (yeast grown in SM containing 3% glycerol–2% galactose, see material an methods) SSC transcripts was readily undetectable in yeasts expressing DCL4 grown in SM containing 3% glycerol–0.1% dextrose (GAL1 repression). Interestingly, we found that in cells in which SSC was co-expressed with DCL4, the quantity of the RNA transcript was reduced by approximately 50% compared to cells in which DCL4 was not present (Fig. 2C), indicating an effective processing of SSC.

RNT1 (the sole *S. cerevisiae* RNaseIII-like protein) is not capable of producing 2-nt 3′ overhangs sRNA duplexes having the chemical features of Dicer products, *i.e.*5′- monophosphates and 3′-hydroxyls. To gain more detailed insight into the composition of the DCL4-mediated processing products of the SSC and DCL4 co-expressing yeast cells, we prepared sRNA libraries with standard protocols and sequenced them using next generation sequence (NGS) analysis by preparing sRNA-seq libraries (see “Materials and methods” section).

Interestingly, with three independent replicates yielding on average more than 11 million reads per experiment, over 70% of the sRNAs were found to originate from the expected double-stranded portion of the SSC transcript (see Fig. 2D and below). While the reads derived predominantly from sRNAs with a length of 21-nt, we also detected considerable quantities of sRNAs with 20- and 22-nt, respectively (Fig. 2E). As mentioned previously, DCL4 has been characterized earlier as an enzyme that is specialized for the production of 21-nt trans acting (ta)-siRNAs. Therefore, the detection of 20-nt and 22-nt sRNAs was unexpected and markedly different from observations with the *S. castellii* DCR1, which was found to process SSC mainly to 23-nt sRNAs [16].

To exclude that these observations were due to DCL4 being active in the heterologous *S. cerevisiae* system, and thus, for example, in the absence of a putative dicing cofactor, we decided to re-analyze the sRNA profiles of the TAS2 transcript in *A. thaliana* Col-0 WT  and *dcl2* and *dcl4* mutant plants, respectively [45]. TAS2 is a non-coding plant transcript, which is efficiently processed by the plant’s DCL4 [13, 49, 50]. We found that the size profile of sRNAs derived from the TAS2 transcript in *Arabidopsis* Col-0 and *dcl2* showed a high similarity to the size profile of sRNAs observed in DCL4-expressing yeast, as a means of high numbers of generated 21-nt and 22-nt sRNAs (Fig. 2F). This finding contrasted with the situation in the *dcl4*-mutant, where we found an accumulation of 22-nt sRNAs (Fig. 2F). Accordingly, this dataset suggests that the DCL4-mediated processing of a dsRNA substrate yields not only 21-nt but also sRNAs of other sizes, namely 22- and 20-nt sRNAs (see “Discussion” section).

Further analysis of sRNA distribution revealed that 20-, 21- and 22-nt siRNAs derived from the entire dsRNA region of SSC (Fig. 2G, upper, middle and lower panels, respectively). While a comprehensive statistical analysis on potential substrate preferences of DCL4 on the SSC RNA substrate remained insignificant, our data showed that the DCL4-catalyzed endonucleolytic processing into the differently sized sRNAs occurred in a highly comparable fashion in the predicted double-stranded region of the SSC hairpin (Additional file 1: Fig. S1) sRNA coverage was significantly lower in the presumably most single-stranded loop region (determined by Fisher’s test, delineated by the vertical red line in Fig. 2G).

### In vivo, DCL4 poorly processes a viral replicase-assisted DI-RNA replicon

**Fig. 3:**
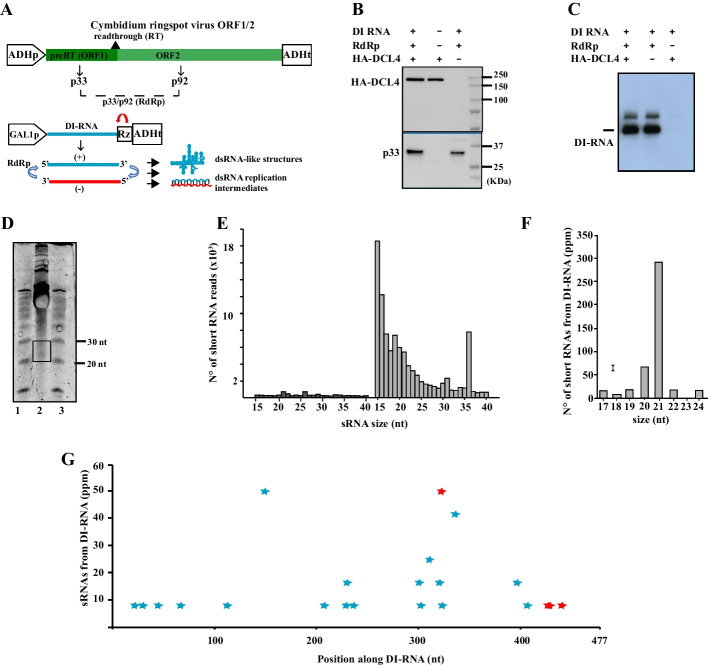
in vivo functionality of recombinant DCL4 in *S. cerevisiae* on a viral replicase-assisted DI-RNA replicon. **A** Schematic representation of expression cassettes used to co-express CymRSV p33/p92 and DI-RNA replicon. CymRSV RNA-dependent RNA polymerase (RdRp) was under the control of the constitutive alcohol dehydrogenase (ADH) promoter and DI-RNA was under the control of the galactose-inducible GAL1 promoter. The arrow (in red) represents the tobacco ringspot virus (TRSV) satellite RNA ribozyme (Rz) [21]. **B** Western blot analysis of whole cell lysates showing HA-DCL4 and/or p33 expression after 24 h growth at 21°C. Detection of HA-DCL4 was performed with a specific anti-HA monoclonal antibody. After stripping, detection of the p33 component of the CymRSV RdRP was performed on the same membrane with a specific polyclonal antiserum. (-) indicating that the corresponding empty plasmid was used for yeast transformation. **C** Northern blot analysis of whole cell lysates showing DI-RNA accumulation and replication. DI-RNA was detected with a specific DIG-labeled RNA probe. (−) indicates that the corresponding empty plasmid was used for yeast transformation. **D** Denaturing PAGE of sRNA-enriched fraction stained with EtBr from yeast cells co-expressing HA-DCL4, CymRSV p33/p92 and DI-RNA replicon (lane 2). 10 bp Ladder in lanes 1 and 3. **E** Size distribution of total sRNAs in cells co-expressing CymRSV p33/p92 and DI-RNA replicon in absence (dark gray) and presence (gray) of HA-DCL4. **F** Size distribution of DI-RNA derived sRNAs. **G** Distribution of the 5′-end nucleotide of sRNAs along the 477-nt long DI-3 RNA. Blue and red refer to ( +) and (−) orientation, respectively

When components of the tombusvirus replicase complex are expressed, DI-RNA transcripts are amplified with the formation of (−) and ( +) strands and the synthesis of head-to-tail dimers[51]. Considering that DEYF is capable of processing both ss- and ds-DI-RNAs into sRNAs in vitro (Fig. 1E), we tested the possibility that DCL4 could process the CymRSV DI-RNA in active replication in vivo*.* For this purpose, we co-expressed the p33 and p92 replicase components and the DI-3 RNA of the tombusvirus CymRSV, as previously reported [21, 52] in the absence and presence of DCL4 (Fig. 3A). Western blot analysis of whole cell lysates showed DCL4 and/or p33 expression after 24 h of growth (Fig. 3B). Also, a massive amount of DI-RNA accumulation could be observed in Northern blot analysis, accordingly (Fig. 3C). The minor upper band above the main RNA types in Fig. 3C have been previously characterized as DI-RNA dimers, which are signs of active and membrane-associated replication events [52]. We have enriched 20- to 30-nt sRNAs from *S. cerevisiae* where the replication of the DI-RNA occurred and the typical sRNA band was visible only in case of co-expression of the DCL4 (Fig. 3D**).** In turn, sequencing libraries were prepared representing the subset of sRNAs with 5′-monophosphates and 3′-hydroxyls, which are the chemical features of DCL4 products. As expected and according to previous studies [16], the presence of DCL4 significantly increased sRNAs from the yeast genome (Fig. 3E). Only a small fraction of siRNAs aligned to the DI-RNA if no mismatches were allowed. It has previously been found that viral replication supports a 3% frequency of single nucleotide variations in DI-RNA progeny [21]. This finding suggests the possibility of single nucleotide variation occurring in sRNAs derived from the DI-RNA progeny. Therefore, we aligned sRNAs to a DI-RNA template allowing one mismatch and obtained a total of 650 per million sRNAs from DI-RNA. The sRNAs derived from DI-RNAs were predominantly 22-nt followed by 21-nt (Fig. 3F). Along the whole 477-nt long DI-RNA, we found only 22 unique sRNAs from the (+) strand and 3 from the (−) strand (Fig. 3G). In the *S. cerevisiae* system, the (+) and (−) strands of the DI-RNA were stabilized cooperatively by the viral replication components [52], the nascent ( +) ssDI-RNAs were associated with membranes and replicase complexes [21] and protected by a proliferation and rearrangement of membranes [53]. All these data show that the DI-RNA actively engaged in replication is not promptly and efficiently processed by DCL4 in yeast.

### 21- and 22-nt sRNAs are associated with sense-antisense transcript loci in DCL4-expressing *S. cerevisiae*

**Fig. 4:**
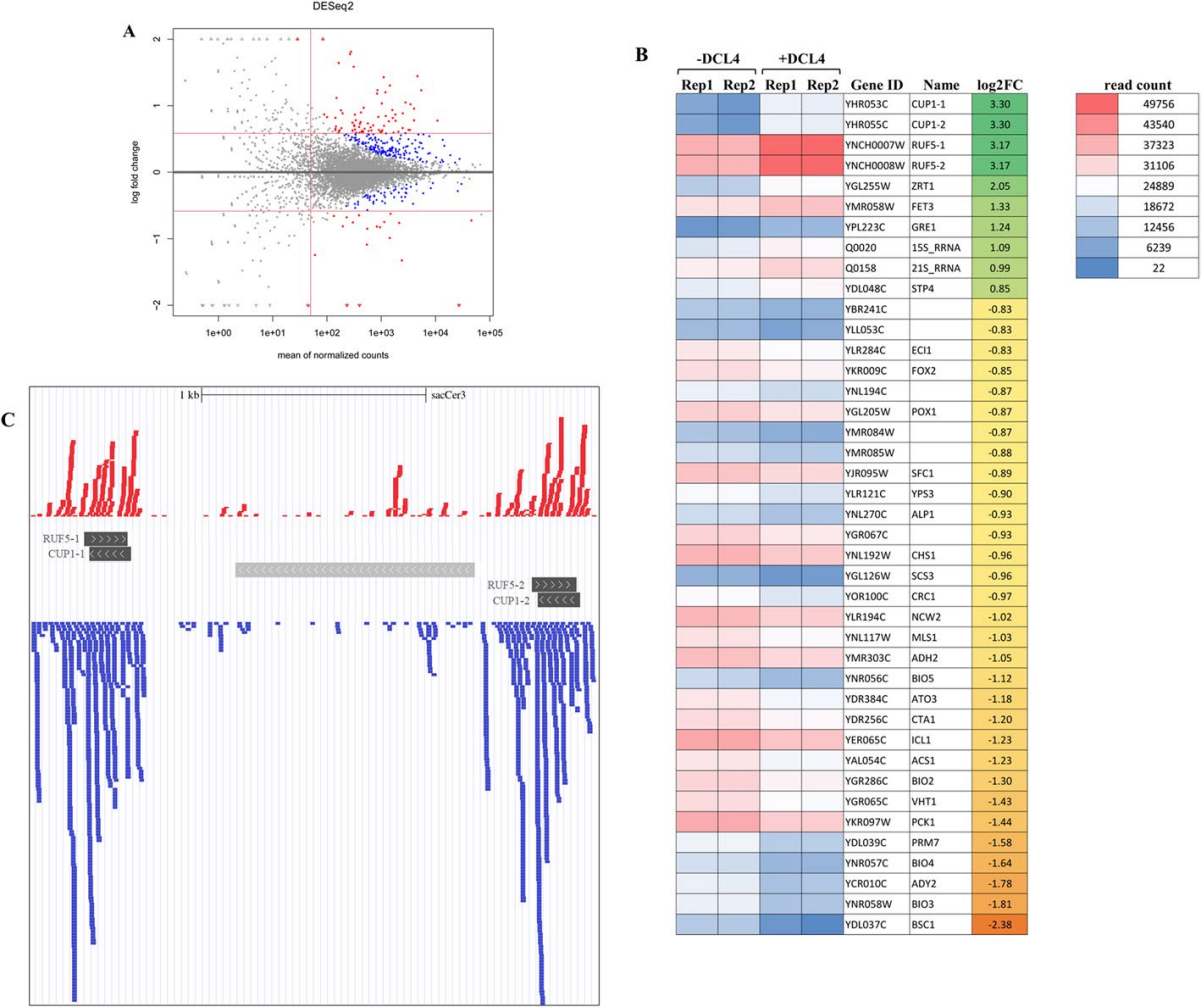
Impact of DCL4 expression in *S. cerevisiae* on transcriptome and on putative sense/antisense RNA loci. **A** Expression of endogenous RNA transcripts in DCL4 expressing and non-expressing *S. cerevisiae*. Plasmid-transformed/non-induced cells were compared with plasmid-transformed copper-induced cells. MA-plots show the distribution of expression fold changes (in log2 scale) as a function of average expression (the baseMean value of DEseq2) of every locus in the compared samples. Positive log2FC values mean that the expression of the given locus was higher when HA-DCL4 was induced, whereas the negative log2 fold change value means that it was lower when HA-DCL4 was induced. Red dots represent expressions changed with an adjusted p-value < 0.05 (5% false discovery rate), absolute Fold Change > 1.5 fnd baseMean > 50. (see Additional file 2: Table S1). **B** Heat map representing the top 40 RNA loci with significant differential expression (adjusted *p*-value < 0.05 and abs(log2(FC)) > 0.585) upon induction of HA-DCL4 expression. **C** Representation of the genomic organization of the CUP1-1 and CUP1-2 clusters. As indicated with the black boxes, the CUP genes are organized in tandem, each with the RUF5-1 and 2 genes organized in antisense direction. Distribution of 21- and 22-nt long DCL4-derived sRNAs of positive (red) and negative (blue) strands. Boxes in gray are other genes associated with the loci of interest

Antisense transcription is recognized as an important inter-kingdom RNA-based regulatory mechanism of gene expression. The transcription of antisense RNAs is part of self-regulatory circuits that allow genes to regulate their own expression. The non-coding transcripts denoted as RNA of Unknown Functions 5 (RUF5-1 and 2) are well-known regulatory and antisense transcripts of genes encoding copper chelation proteins CUP1-1 and 2, respectively [49]. Copper sulfate was added to the culture medium to induce DCL4 expression (see “Materials and methods” section) and, accordingly, CUP1-1 and CUP1-2 were expected to be over-expressed [49]. We performed differential expression analyses with cells in which DCL4 expression was induced by copper and with cells in which it was not. We found 19 significantly over-expressed genes and 67 downregulated genes (Additional file 2: Table S1 and Fig. 4A). As expected, CUP-1 and CUP-2 were among the over-expressed genes. When discovering RUF5 non-coding RNAs, the authors proposed that the CUP1 transcript and the antisense RUF5 RNA could be reciprocally regulated in a stationary phase; however, according to their preliminary results, the RUF5 transcript did not appear to be involved in simple copper-responsive regulation of the CUP1 transcript level [54]. Accordingly, we observed an increase in RUF5 expression associated with an increase in CUP1 expression in the log phase of growth (Fig. 4B). RUF5 and CUP1 co-expressed transcripts could anneal to form dsRNAs, which could be perceived by DCL4. Indeed, upon induction of DCL4, massive amounts of 21- and 22-nt sRNAs were found to be associated with the genomic region containing the CUP1-1/RUF5-1 and CUP1-2/RUF5-2 tandems, with (+) and (−) sense symmetrical peaks. RUF5-1 and 2 non-coding transcripts spanned the CUP1-1 and CUP1-2 coding loci on the antisense strands, respectively (Fig. 4C).

**Fig. 5:**
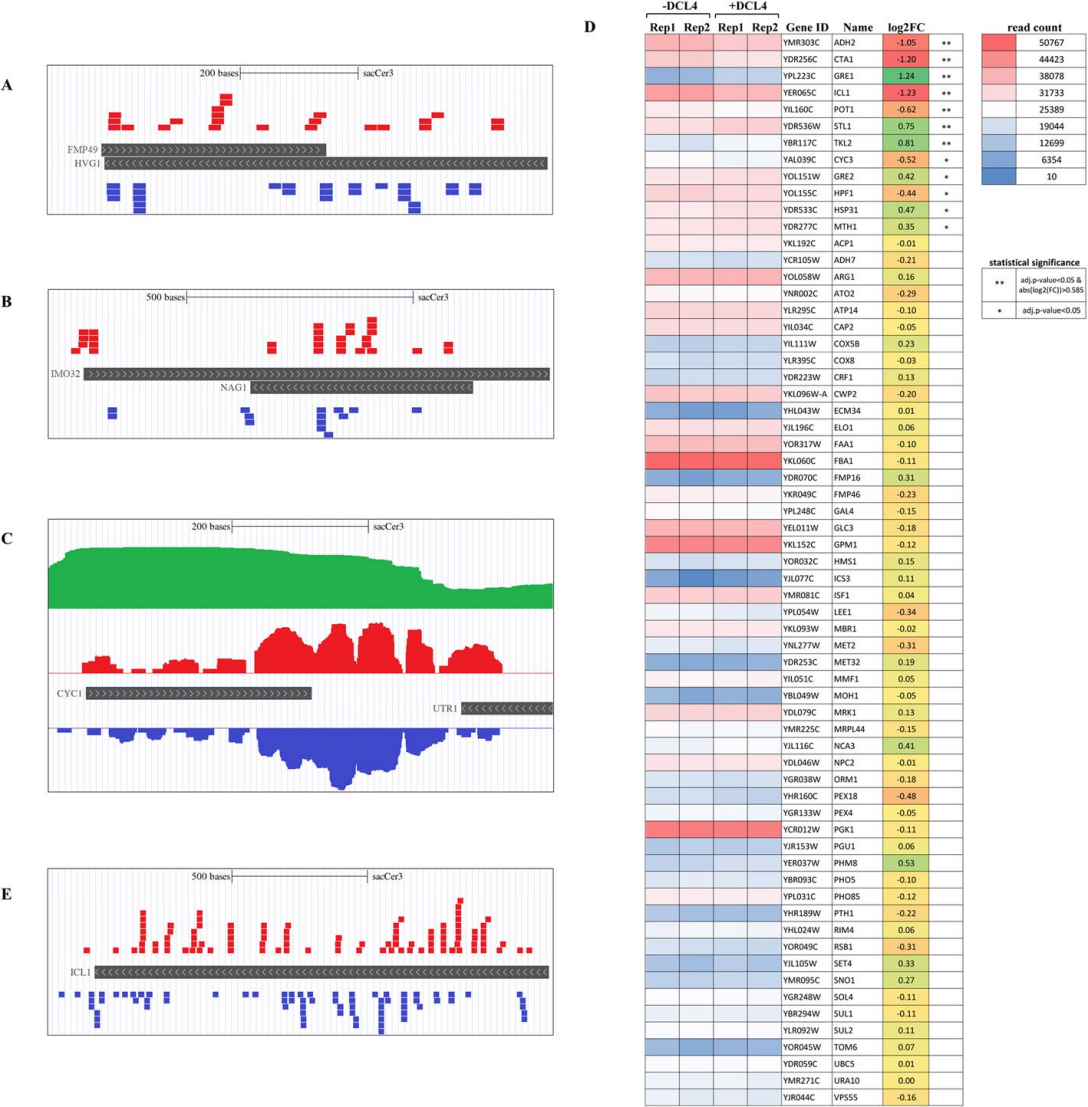
Impact of HA-DCL4 expression in Saccharomyces cerevisiae on annotated and putative antisense transcripts. Representation of the genomic organization and distribution of 21- and 22-nt DCL4-derived sRNAs of FMP49/HGV1 (**A**), IMO32/NAG1 (**B**), CYC1/UTR1 (**C**), and ICL1 (**E**). sRNAs of positive and negative strands are red and blue, respectively. Bars in dark gray are the genes under analysis. Due to the large amount of reads mapped to this locus, positive and negative sRNA reads are represented with a coverage plot, in **C**, and the additional green coverage plot represents RNA transcript from paired ends NGS. **D** Heat map representing the expression of selected putative antisense RNA loci upon induction of HA-DCL4 expression. Significance: **adjusted *p*-value < 0.05 and abs(log2(FC)) > 0.585, *adjusted *p*-value < 0.05

The above evidence prompted us to search for sRNAs of both strands associated with other transcripts that have been sufficiently expressed and previously annotated as sense/antisense on the *S. cerevisiae* genome, (Additional file 3: Table S2). Among these, we identified FMP49/HGV1 and IMO32/NAG1 (Fig. 5A and B, respectively): FMP49 (Found in Mitochondrial Proteome 49) is a conserved mitochondrial protein of unknown functions [55], while HVG1 (Homologous to Vandate Resistance Glycosylation 1) is probably a GDP-mannose transporter 2 [56]; IMO32 (Intermediate cleaved by Mitochondrial Octapeptidyl aminopeptidase 32) is yet another conserved mitochondrial protein of unknown function [57] and overlaps with NAG1 (Nested Antisense Gene 1), encoding a protein that is involved in cell wall biogenesis and confers resistance to cell wall perturbants during thermal stress [58] (Fig. 5B).

Altogether, these results suggest that DCL4 is able to detect and process dsRNAs originating from the co-transcription of antisense transcripts and produces sRNAs from many other overlapping genes. Therefore, we have integrated transcriptome (RNAseq) and sRNA production (sRNAseq) data to search for unknown or sense/antisense transcripts non yet annotated (Additional file 4: Table S3). We have found that the genomic locus encoding mitochondrial Cytochrome C1 (CYC1) produces a large amount of sRNAs of both ( +) and (-) strands able to cover the majority of the transcript, while any antisense gene is annotated in that locus (Fig. 5C). Rather, downstream of CYC1 there is a gene with an opposite orientation, the Unidentified Transcript 1 (UTR1), which encodes an ATP-NADH kinase. A reasonable explanation would be an overlapping transcript synthesis by elongation of mRNAs, as previously described [1]. Accordingly, we have detected a conspicuous amount of 21- and 22-nt sRNAs associated with the RNA transcripts from CYC1 and UTR1 intergenic regions (Fig. 5C, green bars).

SRNAs of 21- and 22-nt and both ( +) and (−) strands were also detected in association with the IsoCitrate Lyase (ICL1) locus. ICL1 is involved in the glyoxylate cycle, and its expression enables the cell to grow on non-fermentable carbon sources (i.e. ethanol). Its expression is known to be de-repressed in the absence of glucose [59]. Expression analysis indicates that the gene is significantly upregulated during the expression of DCL4 (i.e. DCL4 expression culture medium contains a prevalence of non-fermentable carbon sources: 3% glycerol, 0.1% glucose, see “Materials and methods” section) (Additional file 2: Table S1 and Fig. 5D). As shown in Fig. 5E, in this case there is no annotated antisense gene or even a likely extension of the mRNA of a nearby antisense gene. The production of sense/antisense 21- and 22-nt sRNAs is compatible with the presence of not yet annotated antisense transcripts previously detected by a strand-specific RNA sequencing in similar mid-log growth phase [60].

The above evidence prompted us to verify whether the presence of 21- and 22-nt sRNAs is associated with other yet unannotated antisense transcripts, as previously described by Yassour et al. [60]. We have shown that a reasonable amount of sense/antisense sRNAs are associated with the selected loci. In addition, at least six of these (including ICL1) were found to be significantly downregulated in the presence of DCL4, suggesting that the processive capacity of DCL4 is able to negatively regulate the accumulation of dsRNAs similarly to SSC RNA (Fig. 2C).

## Discussion

Arabidopsis crude extracts retain the functionality of DCL4 in processing perfect dsRNAs [61], and similarly we show that the heterologous DCL4 expressed from *S. cerevisiae* is active in processing ss- and ds- defective-interfering RNAs of tomato bushy stunt virus in vitro (Fig. 1G). However, in Arabidopsis, a dsRNA-binding protein, DRB4, is required by DCL4 for the in vitro processing of dsRNAs into 21-nt siRNAs [62]. Immunoprecipitation analyses showed that in vivo DRB4 is only in part associated with DCL4, and that DRB4 absence reduces (but does not abolish) the production of 21-nt siRNAs from plant endogenous dsRNAs (i.e. TAS transcripts); thus, 22-nt-long siRNAs gain visibility in Northern blot analysis [54]. Previous studies have shown that *S. castellii* DCR1 processes target RNAs into mostly 23-nt sRNAs when expressed in *S. cerevisiae*, similarly to the original context [16]. Plant DCL4 has a much more complex domain organization when compared to DCR1 (Additional file 5: Fig. S1), which would ensure specific features leading to the production of 21-nt sRNAs [63]. Note of worthy, when searching for conserved protein domains (https://www.ncbi.nlm.nih.gov/Structure/cdd/cdd.shtml), we could not find neither an analogue to plant DRB4 nor to animal dsRNA-binding proteins in *S. cerevisiae*. Accordingly, we observed the strong capacity of DCL4 to process the predicted dsRNA region of SSC RNA into 21-nt sRNAs followed by 22-nt sRNAs (Fig. 2E). The study by Nakazawa et al. [64] led to postulate that DCL4 is not always associated with DRB4. Therefore, when DCL4 was prevalent (i.e. in *dcl2-4* plants) we found that the size profile of sRNAs from the TAS2 transcript in Arabidopsis paralleled what we found in yeast (Fig. 2F *vs* Figs. 2E and 3F). Our data suggest that DCL4 is functional in *S. cerevisiae* in the absence of additional plant co-factors. Moreover, these findings suggest functionalities of DCL4 in producing 22-nt sRNAs in plants; endogenous sources of dsRNA were originally considered as Pathogen Associated Molecular Patterns and their generation was solely associated with infection by pathogens. However, studies in the past two decades have focused on diverse cellular sources for dsRNAs and their frequent occurrence either during normal physiological processes or upon various types of physiological perturbations. Similar to viral dsRNA, endogenous dsRNAs activate cellular dsRNA sensors [65], such as DCL4 in plants [36, 45, 66], and broad-spectrum as well as cell stress responses via the production of 22-nt siRNAs.

In mammals, studies have failed to detect significant levels of virus-derived siRNAs in several virus/host systems despite the presence of Dicers and Argonautes, which makes mammal cell RNAi compatible. Explanations come from recent studies showing that interferon response in mammalian somatic cells masks dsRNAi, which prevents Dicer sensing [67, 68]. In addition, downstream the translation of viral structural and non-structural proteins, the inhibition of dsRNAi may be further compounded by viral silencing suppressors; indeed, most viral silencing suppressors of mammalian viruses bind dsRNAs [69]. In plants and insects, on the contrary, the amount of vsiRNA invading tissues massively accumulates due to (i) the predominant role of DCLs in sensing dsRNA of viral origin, (ii) the absence of a canonical interferon response, and (iii) the common sRNA-binding action of viral silencing suppressors (in plants). Tombusviruses (family Tombusviridae [70]) are positive-strand RNA viruses and widespread plant pathogens. In tombusvirus infection, DI-RNAs are subviral RNAs incapable of autonomous replication but containing all of the necessary *cis*-acting elements for replication when supported by the viral replicase complex [71]. In *N. benthamiana*, the DI-RNA of Cymbidium ringspot virus (CymRSV) is a potent trigger of RNA silencing because the secondary structure of the molecule is similar to dsRNAs and, considering its ability to accumulate in infected tissues, is a preferred substrate of antiviral DCLs, including DCL4 [50].

Yeast systems have been exploited for studying the replication of tombusvirus DI-RNAs. Whether dsRNA replication intermediates are triggers of vsiRNA production is still an open question in both animal and plant virus systems. In the *S. cerevisiae* system the (+) and (−) strands of the DI-RNA are stabilized cooperatively by the viral replication components [72] and the nascent (+) ssDI-RNAs are associated with membranes and replicase complexes [21] and protected by a proliferation of membranes [73]. In the RNAi depleted system, our results clearly show that a yeast enriched fraction of the plant DCL4 was able to process transcribed viral ss- and dsRNA (Fig. 1G) in vitro. In vivo, the plant DCL4 processed dsRNA hairpins into sRNAs (Fig. 2E), and few vsiRNAs were from the replicase assisted DI-RNA (Fig. 3G). These findings suggest that the plant DCL4 is not able to access the niches of the replication machinery where the viral RNAs and derivatives are replicated in a protected environment, as is widely accepted in virus-infected mammalian cells. Our observations are consistent with the massive accumulation of vsiRNAs in plants and the supremacy of the production of vsiRNAs from the (+) strand over those from the (−) strand [47, 74]. Indeed, antiviral DCLs process free (+) stranded viral RNAs, i.e. those viral RNAs released into the cytoplasm from the replication machinery and invading the host plant.

Novel and efficacious antiviral RNAi research should take these evidence into account (reviewed by [75]). For instance, treatment with sRNAs of both mammalian and plant cells prior to viral infection can in principle prevent the assembly of the Dicer-resistant replication niches and pathogenesis. After the recent discovery and characterization of the budding yeast pathway [20], RNAi could be used as a tool to silence genes in *S. cerevisiae, S. castellii*, and presumably other budding yeasts. In the present study we extended previous applications of heterologous Dicers in *S. cerevisiae* and the potential to fully reproduce the ruler-cutting function of DCL4 with no lethal effects or abnormalities for the recipient. In accordance with the previous observation on the processivity of *S. castellii* DCR1, we observed a massive amount of sRNA by DCL4 from the yeast retrotransposon *Ty-1* (Additional file 5: Fig. S2). However, endogenous transcripts were impacted (Figs. 4A, B and 5D), illustrating the ability of DCL4 not only to process but also and to downregulate targets in the case of the SSC RNA (Fig. 2C). The plant DCL4 expressed in yeast readily recognized endogenous dsRNAs and was able to confirm yet unannotated antisense transcripts [60] (Fig. 4C), or even reveal new transcripts (Fig. 5A–C and E). Among these loci at least four, FMP49/HGV1, IMO32/NAG1, CYC1/UTR1, and ICL1, code for mitochondrial and nuclear proteins from Watson and Crick strands, respectively (Fig. 5A and B). These findings promote the concept that the co-transcription of sense/antisense RNAs can be a conserved RNA-based mechanism mediating nuclear-mitochondria communication. The analyses conducted in this study are based on the growth of a laboratory yeast strain under specific culture conditions in terms of sugars and salts for protein induction. Nevertheless, the heterologous DCL4 could be a potent tool to reveal sense-/antisense-coupled RNAs either in the non-physiological expression of the gene of interest (e.g., the galactose-glucose, copper sulfate system) or under stress conditions of growth.

### Supplementary Information

**Additional file 1: Figure S1.** RNAfold results in the case of aberrant SSC transcripts.

**Additional file 2: Table S1.** Differentially expressed genes (DESeq2). DESeq2 results for differential expression analysis of RNA-Seq data. Legend: baseMean (mean read count) is highlighted in yellow if > 50; log2FC > 0.585 (corresponding to FC > 1.5) is colored in red, log2FC < -0.585 is blue; significant p-value < 0.05 is green.

**Additional file 3: Table S2.** Sense-antisense annotated genes derived sRNAs. Results are shown for each replicate (21_sample1, 25_sample2, 30_sample3, highlighted in different colors in the "sample" column). Genes highlighted in yellow are represented in the paper: RUF5-1/CUP1-1 and RUF5-2/CUP1-2 (Fig. 4); FMP49/HGV1 and IMO32/NAG1 (Fig. 5).

**Additional file 4: Table S3.** Sense-antisense sRNAs derived from single stranded annotated genes. Results are shown for each replicate (21_sample1, 25_sample2, 30_sample3, highlighted in different colors in the "sample" column). Genes highlighted in yellow are represented in the paper: CYC1, UTR1 and ICL1 (Fig. 5).

**Additional file 5: Figure S1.** Domain architectures of *N. benthamiana* DCL4 and of *S. castellii* DCR1. PAZ: Piwi/Argonaute Zwille domain; dsRBM: double-stranded RNA binding motif. **Figure S2.** DCL4-dependent 21- and 22-nt sRNAs from S. cerevisiae retrotransposon Ty1. sRNA-Seq analysis of *S. cerevisiae* Ty1 elements. The 21- and 22-nt sRNAs to a consensus Ty1 element (Ty1 retrotransposon B10 were plotted (sense, blue; antisense, purple). The schematic shows the proviral Ty1 reference (Acc. N. M18706.1) with long terminal repeats (LTRs) and *gag*/*pol* expression organization of mRNA.

**Additional file 6.** Replicates Raw Western blots and nucleic acid gels. Replicates Raw Western blots and nucleic acid gels relative to the images at A) Fig. 1B, expression of HADCL4 at different temperatures in *S. cerevisiae*, B) Fig. 1D, enrichment of HADCL4, C) Fig. 1E*, *in vitro functionality of yeast crude extract or DEYF in processing Tombusviruses dsRNAs in vitro transcribed, D) Fig. 3B, coexpression of tombusvirus replicase p33 and HADCL4 in yeast, E) Fig. 3C, Northern blot analysis of tombusvirus DI-RNA replication in yeast, F) Fig. 3C Denaturing PAGE of sRNA-enriched fraction stained with EtBr from yeast cells co-expressing HA-DCL4, CymDSV p33/p92 and DI-RNA.

## Data Availability

BAM alignment files loaded in the UCSC browser are stored in the Figshare project 157284 (https://figshare.com/projects/Exogenous_and_endogenous_sRNAs_derived_from_plant_Dicer-like_4_in_the_RNAi-depleted_yeast_Saccharomyces_cerevisiae/157284). Read alignments plotted in Figs. 4 and 5 are available as public sessions in UCSC genome browser at the following URL: https://genome-euro.ucsc.edu/s/cnr.itb.ba/sacCer3_2023.

## Abbreviations

AGO: Argonate
ADH1: Alcohol dehydrogenase 1
BYL: BY-2 protoplasts lysate
cDNA: Complementary DNA
CUP1: Copper chelation protein 1
CYC1: Cytochrome C
CymRSV: Cymbidium ringspot virus
DCLs: RNase III Dicer-like proteins
DDT: Dithiothreitol
DEYF: DCL4-enriched yeast fraction
DI-RNA: Defective-interfering RNA
dsRNA: Double-stranded RNA
FMP49: Found in Mitochondrial Proteome 49
GAL1: Galactose-inducible
GFP: Green fluorescent protein
HA: Hemagglutinin
HVG1: Homologous to Vandate Resistance Glycosylation
ICL1: IsoCitrate Lyase 1
IMO32: Intermediate cleaved by Mitochondrial Octapeptidyl aminopeptidase 32
kDa: KiloDalton
LTR: Long terminal repeats
miRNA: MicroRNA
NAG1: Nested Antisense Gene 1
NGS: Next generation sequence
nt: Nucleotides
PAZ: Piwi/Argonaute Zwille domain;
dsRBM: Double-stranded RNA binding motif
pha-siRNAs: Phased siRNAs
ppm: Part per million
RdRp: RNA-dependent RNA polymerase
RNA: Ribonucleic acid
RNAi: RNA interference
RNAseq: RNA sequencing
RNT1: Ribonuclease III
RUF5: RNA of Unknown Functions 5
Rz: RNA ribozyme
siRNA: Short interfering RNA
SM: Selective medium
sRNA: Short RNA
SSC: Strong silencing construct
ssRNA: Single strand RNA
ta-siRNA: Trans-acting siRNA
TAS transcrip: Trans Acting Small RNA
TRSV: Tobacco ringspot virus
UTR1: Unidentified Transcript 1
vsiRNA: SiRNA of viral origin
