# Supplementary material for: Exogenous and endogenous dsRNAs perceived by plant Dicer-like 4 protein in the RNAi-depleted cellular context
Source: Cell Mol Biol Lett. 2023 Aug 7;28:64. doi: 10.1186/s11658-023-00469-2 (PMC10405411; doi:10.1186/s11658-023-00469-2)

**Additional File 1: Fig. S1. RNAfold results in the case of aberrant SSC transcripts.**

RNAfold run with default parameters on the web application:

<http://rna.tbi.univie.ac.at/cgi-bin/RNAWebSuite/RNAfold.cgi>

Example of transcription from 1 to 355

(the portion including only the 5’ complementary portion and the loop)


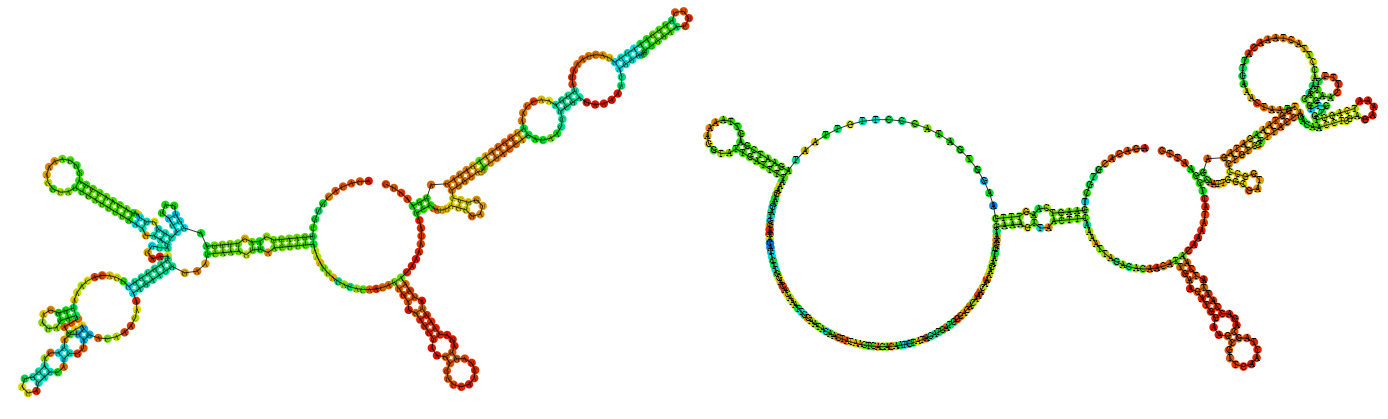


**MFE secondary structure                                                  Centroid secondary structure**

Example of transcription from 1 to 400


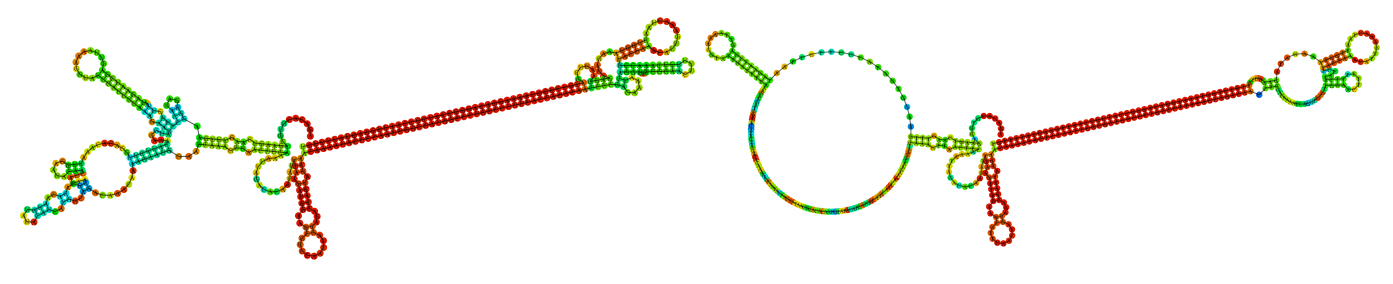


Example of transcription from 1 to 500


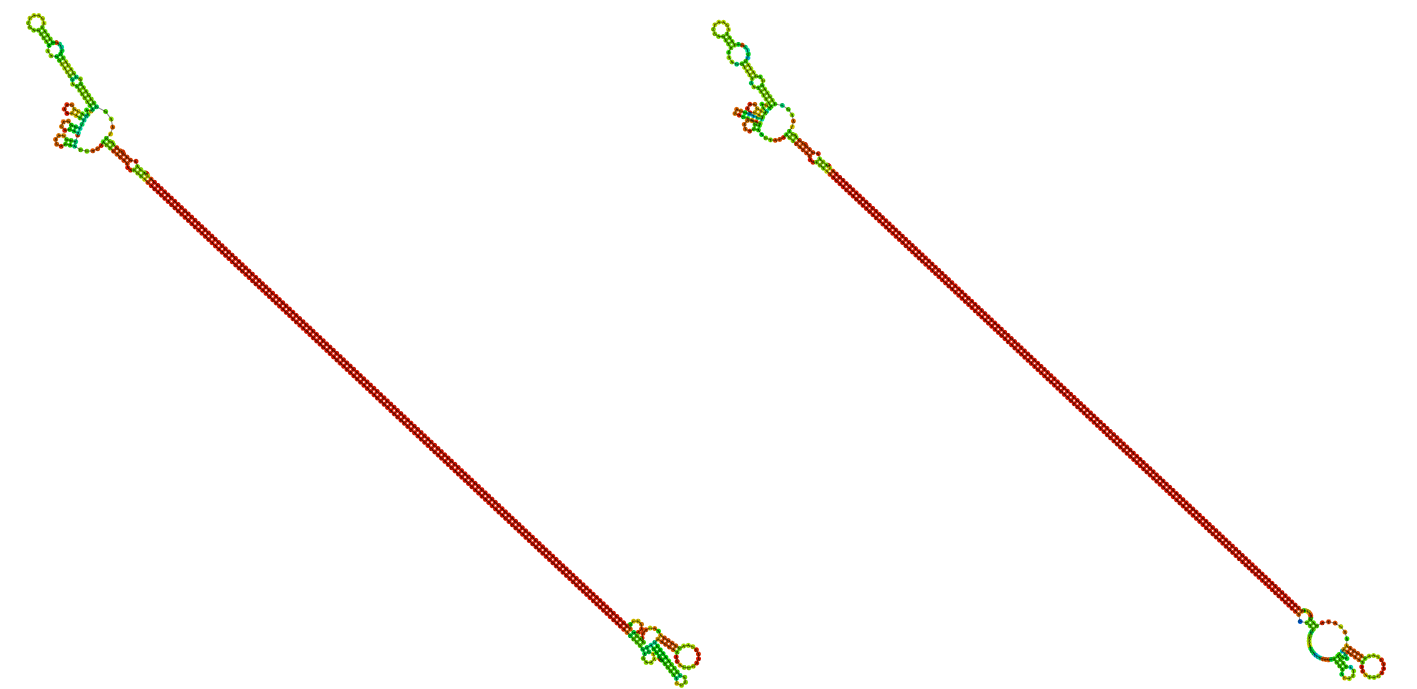

Supplement: Supplementary file 1 — Additional file 1: Figure S1. RNAfold results in the case of aberrant SSC transcripts. [file 11658_2023_469_MOESM1_ESM.docx]
