# Supplementary material for: Exogenous and endogenous dsRNAs perceived by plant Dicer-like 4 protein in the RNAi-depleted cellular context
Source: Cell Mol Biol Lett. 2023 Aug 7;28:64. doi: 10.1186/s11658-023-00469-2 (PMC10405411; doi:10.1186/s11658-023-00469-2)
Supplement: Supplementary file 5 — Additional file 5: Figure S1. Domain architectures of N. benthamiana DCL4 and of S. castellii DCR1. PAZ: Piwi/Argonaute Zwille domain; dsRBM: double-stranded RNA binding motif. Figure S2. DCL4-dependent 21- and 22-nt sRNAs from S. cerevisiae retrotransposon Ty1. sRNA-Seq analysis of S. cerevisiae Ty1 elements. The 21- and 22-nt sRNAs to a consensus Ty1 element (Ty1 retrotransposon B10 were plotted (sense, blue; antisense, purple). The schematic shows the proviral Ty1 reference (Acc. N. M18706.1) with long terminal repeats (LTRs) and gag/pol expression organization of mRNA. [file 11658_2023_469_MOESM5_ESM.docx]

**Additional File 5: Fig. S1. Domain architectures of *N. benthamiana* DCL4 (BankIt2712565 NbDCL4 OR12626) and of *S. castellii* DCR1. PAZ: Piwi/Argonaute Zwille domain; dsRBM: double-stranded RNA binding motif.**

**Additional File 5: Fig. S2. DCL4-dependent 21- and 22-nt sRNAs from *S. cerevisiae* retrotransposon Ty1.**

**Consensus sequence of ScTY1**

Saccharomyces cerevisiae Ty1-H3 gene

Saccharomyces cerevisiae Ty1-H3 gene:

LTRs;

mRNA start; mRNA end; TYA gene start/end; TYB gene start/end

TGTTGGAATAGAAATCAACTATCATCTACTAACTAGTATTTACATTACTAGTATATTATCATATACGGTGTTAGAAGATGACGCAAATGATGAGAAATAGTCATCTAAATTAGTGGAAGCTGAAACGCAAGGATTGATAATGTAATAGGATCAATGAATATAAACATATAAAATGATGATAATAATATTTATAGAATTGTGTAGAATTGCAGATTCCATTTTGAGGATTCCTATATCCTCGAGGAGAACTTCTAGTATATTCTGTATACCTAATATTATAGCCTTTATCAACAATGGAATCCCAACAATTATCTCAACATTCACCCAATTCTCATGGTAGCGCCTGTGCTTCGGTTACTTCTAAGGAAGTCCACACAAATCAAGATCCGTTAGACGTTTCAGCTTCCAAAACAGAAGAATGTGAGAAGGCTTCCACTAAGGCTAACTCTCAACAGACAACAACACCTGCTTCATCAGCTGTTCCAGAGAACCCCCATCATGCCTCTCCTCAACCTGCTTCAGTACCACCTCCACAGAATGGGCCGTACCCACAGCAGTGCATGATGACCCAAAACCAAGCCAATCCATCTGGTTGGTCATTTTACGGACACCCATCTATGATTCCGTATACACCTTATCAAATGTCGCCTATGTACTTTCCACCTGGGCCACAATCACAGTTTCCGCAGTATCCATCATCAGTTGGAACGCCTCTGAGCACTCCATCACCTGAGTCAGGTAATACATTTACTGATTCATCCTCAGCGGACTCTGATATGACATCCACTAAAAAATATGTCAGACCACCACCAATGTTAACCTCACCTAATGACTTTCCAAATTGGGTTAAAACATACATCAAATTTTTACAAAACTCGAATCTCGGTGGTATTATTCCGACAGTAAACGGAAAACCCGTACGTCAGATCACTGATGATGAACTCACCTTCTTGTATAACACTTTTCAAATATTTGCTCCCTCTCAATTCCTACCTACCTGGGTCAAAGACATCCTATCCGTTGATTATACGGATATCATGAAAATTCTTTCCAAAAGTATTGAAAAAATGCAATCTGATACCCAAGAGGCAAACGACATTGTGACCCTGGCAAATTTGCAATATAATGGCAGTACACCTGCAGATGCATTTGAAACAAAAGTCACAAACATTATCGACAGACTGAACAATAATGGCATTCATATCAATAACAAGGTCGCATGCCAATTAATTATGAGAGGTCTATCTGGCGAATATAAATTTTTACGCTACACACGTCATCGACATCTAAATATGACAGTCGCTGAACTGTTCTTAGATATCCATGCTATTTATGAAGAACAACAGGGATCGAGAAACAGTAAACCTAATTACAGGAGAAATCCGAGTGATGAGAAGAATGATTCTCGCAGCTATACGAATACAACCAAACCCAAAGTTATAGCTCGGAATCCTCAAAAAACAAATAATTCGAAATCGAAAACAGCCAGGGCTCACAATGTATCCACATCTAATAACTCTCCCAGCACGGACAACGATTCCATCAGTAAATCAACTACTGAACCGATTCAATTGAACAATAAGCACGACCTTCATCTTAGGCCAGAAACTTACTGAATCTACAGTAAATCATACTAATCATTCTGATGATGAACTCCCTGGACACCTCCTTCTCGATTCAGGAGCATCACGAACCCTTATAAGATCTGCTCATCACATACACTCAGCATCATCTAATCCTGACATAAACGTAGTTGATGCTCAAAAAAGAAATATACCAATTAACGCTATTGGTGACCTACAATTTCACTTCCAGGACAACACCAAAACATCAATAAAGGTATTGCACACTCCTAACATAGCCTATGACTTACTCAGTTTGAATGAATTGGCTGCAGTAGATATCACAGCATGCTTTACCAAAAACGTCTTAGAACGGTCTGACGGCACTGTACTTGCACCTATCGTAAAATATGGAGACTTTTACTGGGTATCTAAAAAGTACTTGCTTCCATCAAATATCTCCGTACCCACCATCAATAATGTCCATACAAGTGAAAGTACACGCAAATATCCTTATCCTTTCATTCATCGAATGCTTGCGCATGCCAATGCACAGACAATTCGATACTCACTTAAAAATAACACCATCACGTATTTTAACGAATCAGATGTCGACTGGTCTAGTGCTATTGACTATCAATGTCCTGATTGTTTAATCGGCAAAAGCACCAAACACAGACATATCAAAGGTTCACGACTAAAATACCAAAATTCATACGAACCCTTTCAATACCTACATACTGACATATTTGGTCCAGTTCACAACCTACCAAATAGTGCACCATCCTATTTCATCTCATTTACTGATGAGACAACAAAATTCCGTTGGGTTTATCCATTACACGACCGTCGCGAGGACTCTATCCTCGATGTTTTTACTACGATACTAGCTTTTATTAAAAACCAGTTTCAGGCCAGTGTCTTGGTTATACAAATGGACCGTGGTTCTGAGTATACTAACAGAACTCTCCATAAATTCCTTGAAAAAAATGGTATAACTCCATGCTATACAACCACAGCGGATTCCCGAGCACATGGAGTCGCTGAACGGCTAAACCGTACCTTATTAGATGACTGCCGTACTCAACTGCAATGTAGTGGTTTACCGAACCATTTATGGTTCTCTGCAATCGAATTTTCTACTATTGTGAGAAATTCACTAGCTTCACCTAAAAGCAAAAAATCTGCAAGACAACATGCTGGCTTGGCAGGACTTGATATCAGTACTTTGTTACCTTTCGGTCAACCTGTTATCGTCAATGATCACAACCCTAACTCCAAAATACATCCTCGTGGCATCCCAGGCTACGCTCTACATCCGTCTCGAAACTCTTATGGATATATCATCTATCTTCCATCCTTAAAGAAGACAGTAGATACAACTAACTATGTTATTCTTCAGGGCAAGGAATCCAGATTAGATCAATTCAATTACGACGCACTCACTTTCGATGAAGACTTAAACCGTTTAACTGCTTCATATCATTCGTTCATTGCGTCAAATGAGATCCAAGAATCCAATGATCTTAACATAGAATCTGACCATGACTTCCAATCCGACATTGAACTACATCCTGAGCAACCGAGAAATGTCCTTTCAAAAGCTGTGAGTCCAACCGATTCCACACCTCCGTCAACTCATACTGAAGATTCGAAACGTGTTTCTAAAACCAATATTCGCGCACCCAGAGAAGTTGACCCCAACATATCTGAATCTAATATTCTTCCATCAAAGAAGAGATCTAGCACCCCCCAAATTTCCAATATCGAGAGTACCGGTTCGGGTGGTATGCATAAATTAAATGTTCCTTTACTTGCTCCCATGTCCCAATCTAACACACATGAGTCGTCGCACGCCAGTAAATCTAAAGATTTCAGACACTCAGACTCGTACAGTGAAAATGAGACTAATCATACAAACGTACCAATATCCAGTACGGGTGGTACCAACAACAAAACTGTTCCGCAGATAAGTGACCAAGAGACTGAGAAAAGGATTATACACCGTTCACCTTCAATCGATGCTTCTCCACCGGAAAATAATTCATCGCACAATATTGTTCCTATCAAAACGCCAACTACTGTTTCTGAACAGAATACCGAGGAATCTATCATCGCTGATCTCCCACTCCCTGATCTACCTCCAGAATCTCCTACCGAATTCCCTGACCCATTTAAAGAACTCCCACCGATAAATTCTCGTCAAACTAATTCCAGTTTGGGTGGTATTGGTGACTCTAATGCCTATACTACTATCAACAGTAAGAAAAGATCATTAGAAGATAATGAAACTGAAATTAAGGTATCACGAGACACATGGAATACTAAGAATATGCGTAGTTTAGAACCTCCGAGATCGAAGAAACGAATTCACCTGATTGCAGCTGTAAAAGCAGTAAAATCAATCAAACCAATACGGACAACCTTACGATACGATGAGGCAATCACCTATAATAAAGATATTAAAGAAAAAGAAAAATATATCGAGGCATACCACAAAGAAGTCAATCAACTGTTGAAGATGAAAACTTGGGACACTGACGAATATTATGACAGAAAAGAAATAGACCCTAAAAGAGTAATAAACTCAATGTTTATCTTCAACAAGAAACGTGACGGTACTCATAAAGCTAGATTTGTTGCAAGAGGTGATATTCAGCATCCTGACACTTACGACTCAGGCATGCAATCCAATACCGTACATCACTATGCATTAATGACATCCCTGTCACTTGCATTAGACAATAACTACTATATTACACAATTAGACATATCTTGGCATATTTGTATGCAGACATCAAAGAAGAATTATACATAAGACCTCCACCACATTTAGGAATGAATGATAAGTTGATACGTTTGAAGAAATCACTTTATGGATTGAAACAAAGTGGAGCGAACTGGTACGAAACTATCAATCATACCTGATACAACAATGTGGTATGGAAGAAGTTCGTGGATGGTCATGCGTATTTAAAAACAGTCAAGTGACAATTTGTTTATTCGTAGATGATATGGTATTGTTTAGCAAAAATCTAAATTCAAACAAAAGAATTATAGAGAAGCTTAAGATGCAATACGACACCAAGATTATAAATCTAGGCGAAAGTGATGAGGAAATTCAATTGACATACTTGGCTTAGAAATCAAATATCAAAGAGGTAAATACATGAAATTAGGTATGGAAAACTCATTAACTGAGAAAATACCCAAATTAAACGTACCTTTGAATCCAAAAGGAAGAAAACTTAGCGCTCCAGGTCAACCAGGTCTTTATATAGACCAGGATGAACTAGAAATAGATGAAGATGAATACAAAGAGAAGGTACATGAAATGCAAAAGTTGATTGGTCTAGCTTCATATGTTGGATATAAATTTAGATTTGACTTACTATACTACATCAACACACTTGCTCAACATATACTATTCCCCTCTAGGCAAGTTTTAGACATGACATATGAGTTGATACAATTCATGTGGGACACTAGAGATAAACAACTGATATGGCACAAAAACAAACCTACCGAGCCAGATAATAAACTAGTCGCAATAAGTGATGCTTCGTATGGCAACCAACCGTATTATAAATCACAAATTGGCAACATATATTTACTTAATGGAAAGGTAATTGGAGGAAAGTCCACCAAGGCTTCATTAACATGTACTTCAACTACGGAAGCAGAAATACACGCGATAAGTGAATCTGTCCCATTATTAAATAATCTAAGTTACCTGATACAAGAACTTAACAAGAAACCAATTATTAAAGGCTTACTTACTGATAGTAGATCAACGATCAGTATAATTAAGTCTACAAATGAAGAGAAATTTAGAAACAGATTTTTTGGCACAAAGGCAATGAGACTTAGAGATGAAGTATCAGGTAATAATTTATACGTATACTACATCGAGACCAAGAAGAACATTGCTGATGTGATGACAAAACCTCTTCCGATAAAAACATTTAAACTATTAACTAACAAATGGATTCATTAGATCTATTACATTATGGGTGGTATGTTGGAATAGAAATCAACTATCATCTACTAACTAGTATTTACATTACTAGTATATTATCATATACGGTGTTAGAAGATGACGCAAATGATGAGAAATAGTCATCTAAATTAGTGGAAGCTGAAACGCAAGGATTGATAATGTAATAGGATCAATGAATATAAACATATAAAATGATGATAATAATATTTATAGAATTGTGTAGAATTGCAGATTCCCTTTTATGGATTCCTAAATCCTTGAGGAGAACTTCTAGTATATTCTGTATACCTAATATTATAGCCTTTATCAACAATGGAATCCCAACAATTATCTCAACATTCACCCATTTCTCA
