# Supplementary material for: Exogenous and endogenous dsRNAs perceived by plant Dicer-like 4 protein in the RNAi-depleted cellular context
Source: Cell Mol Biol Lett. 2023 Aug 7;28:64. doi: 10.1186/s11658-023-00469-2 (PMC10405411; doi:10.1186/s11658-023-00469-2)

Additional file 6: Replicates Raw Western blots and nucleic acid gels.

Replicates Raw Western blots and nucleic acid gels relative to the images at **A)** Fig 1B, expression of HADCL4 at different temperatures in *S. cerevisiae*, **B)** Fig. 1E, enrichment of HADCL4, **C)** Fig. 1F*, in vitro* functionality of yeast crude extract or DEYF in processing Tombusviruses *in vitro* transcribed dsRNAs, **D)** Fig. 3B, coexpression of tombusvirus replicase p33 and HADCL4 in yeast, **E)** Fig. 3C, Northern blot analysis of tombusvirus DI-RNA replication in yeast, **F)** Fig. 3D Denaturing PAGE of sRNA-enriched fraction stained with EtBr from yeast cells co-expressing HA-DCL4, CymRSV p33/p92 and DI-RNA.

A)

Chemioluminescence **Ponceau Staining**

selected for main image

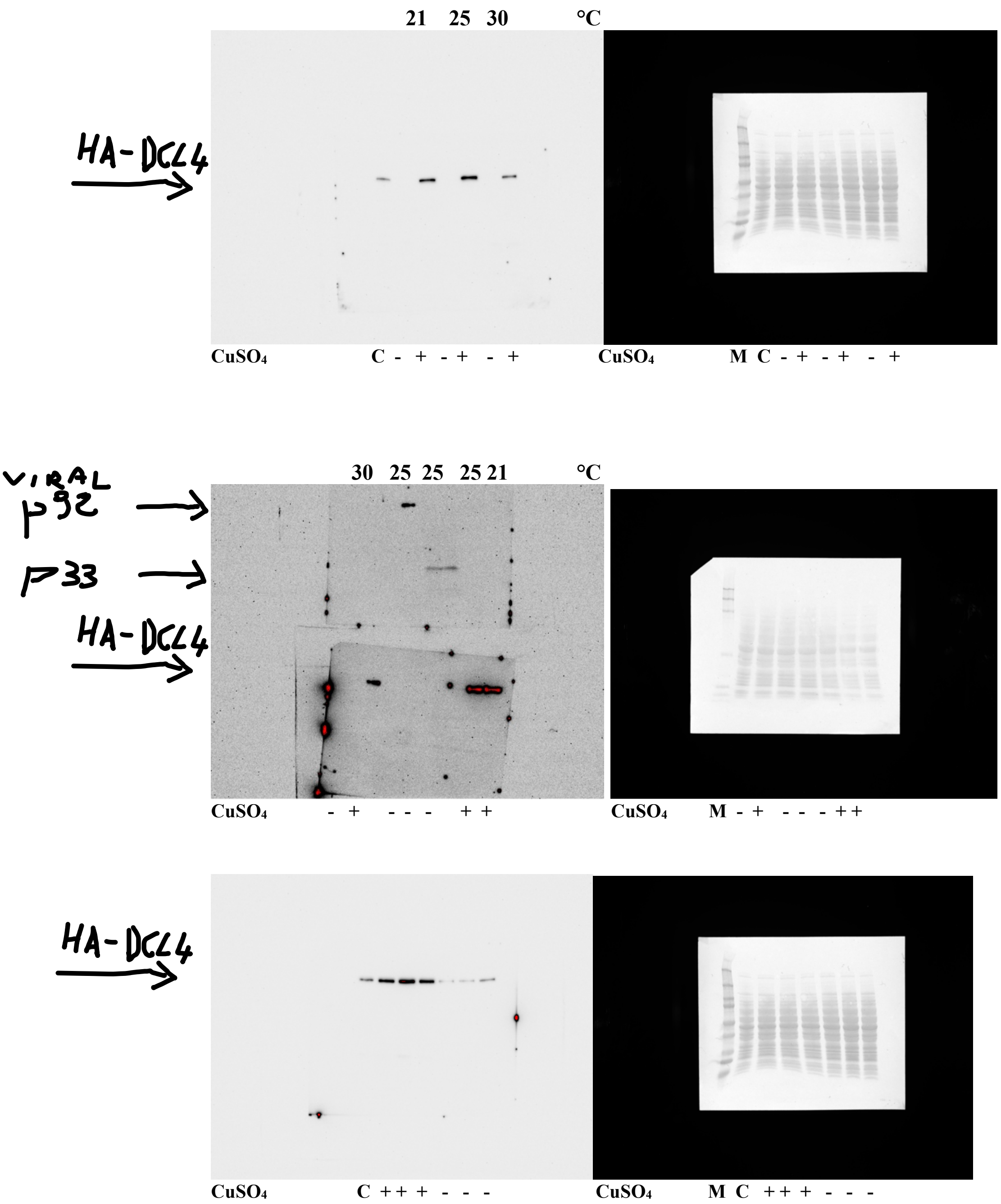

B)

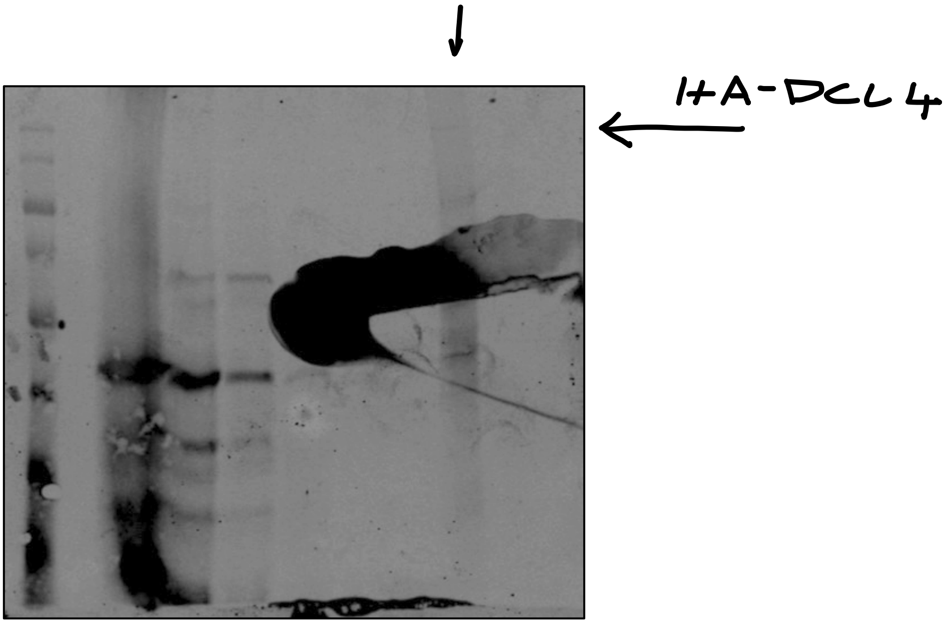

Selected for the main image

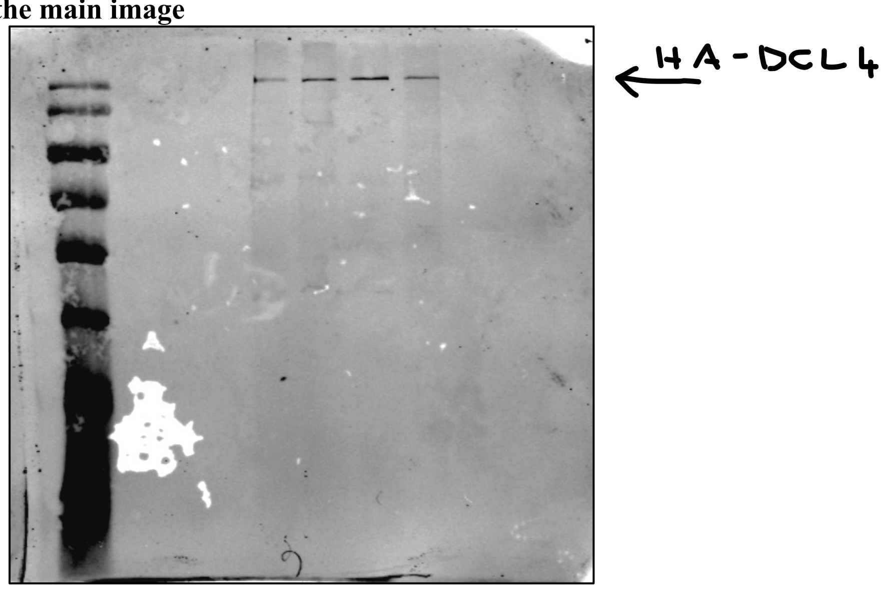

C)

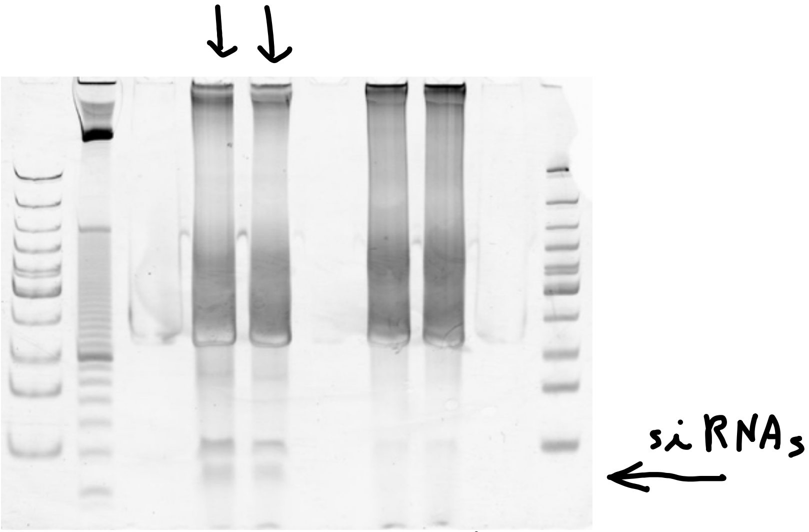

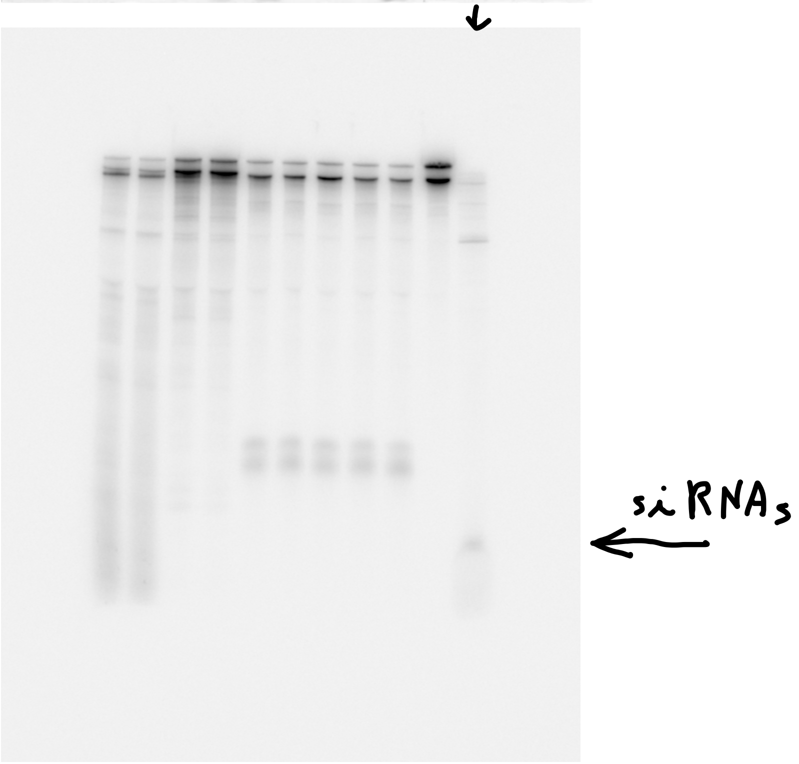

Selected for the main image

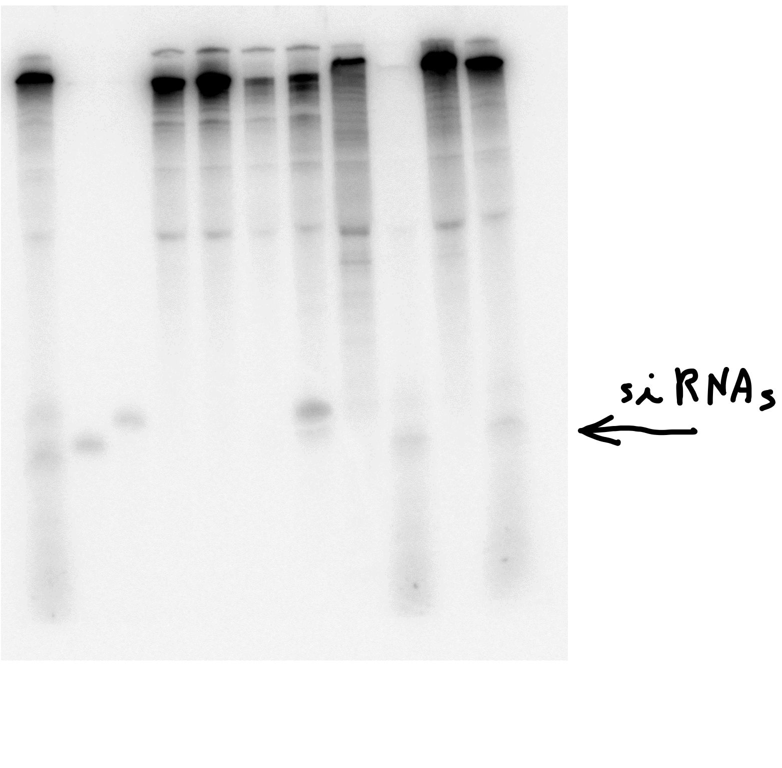

D)

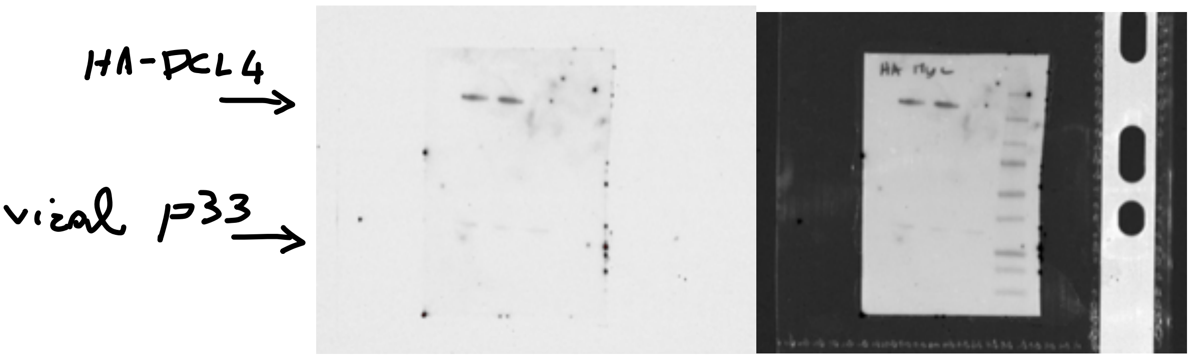

HA-DCL4	+	+	-	M
Myc-p33	+	+	+	M

HA-DCL4	+	+	-	M
Myc-p33	+	+	+	M

Selected for the main image

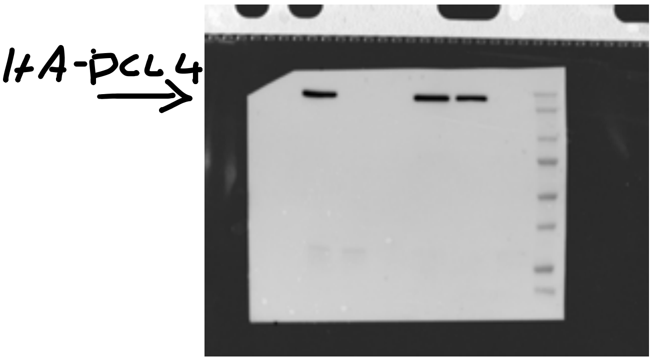

HA-DCL4	+	-	-	+	+	-	M
Myc-p33	-	-	-	+	-	+	M

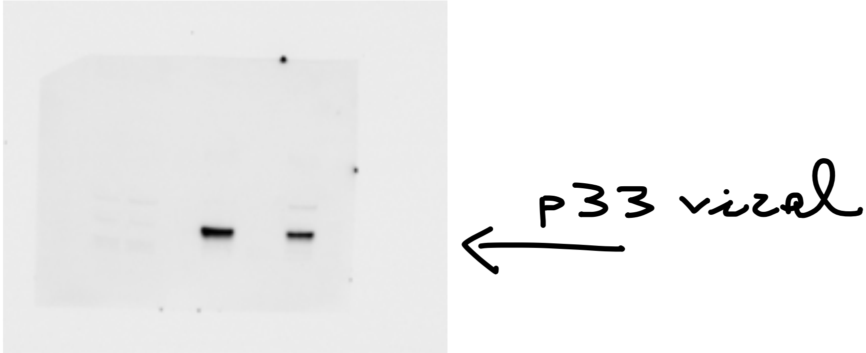

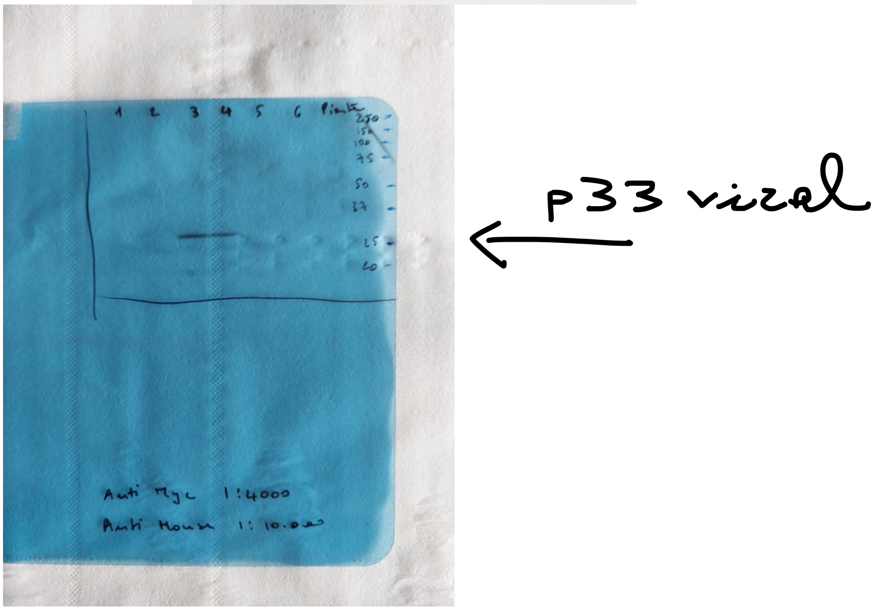

E)
Selected for the main image

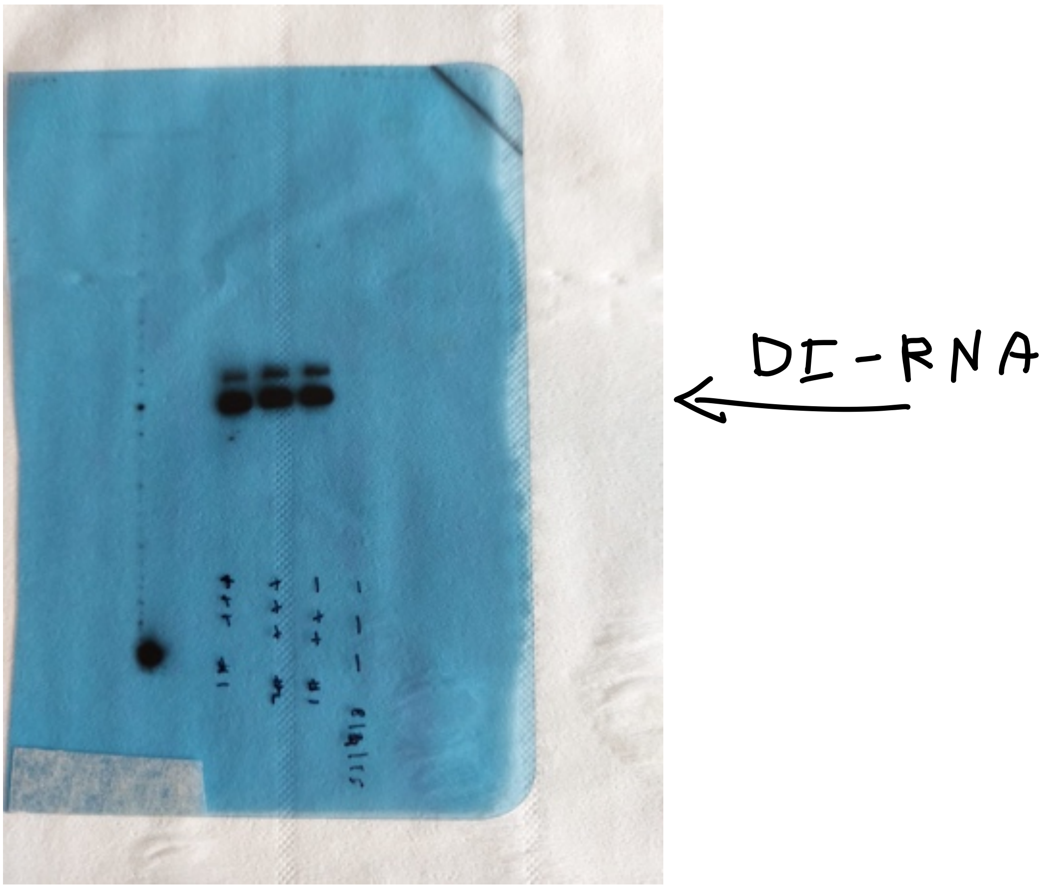

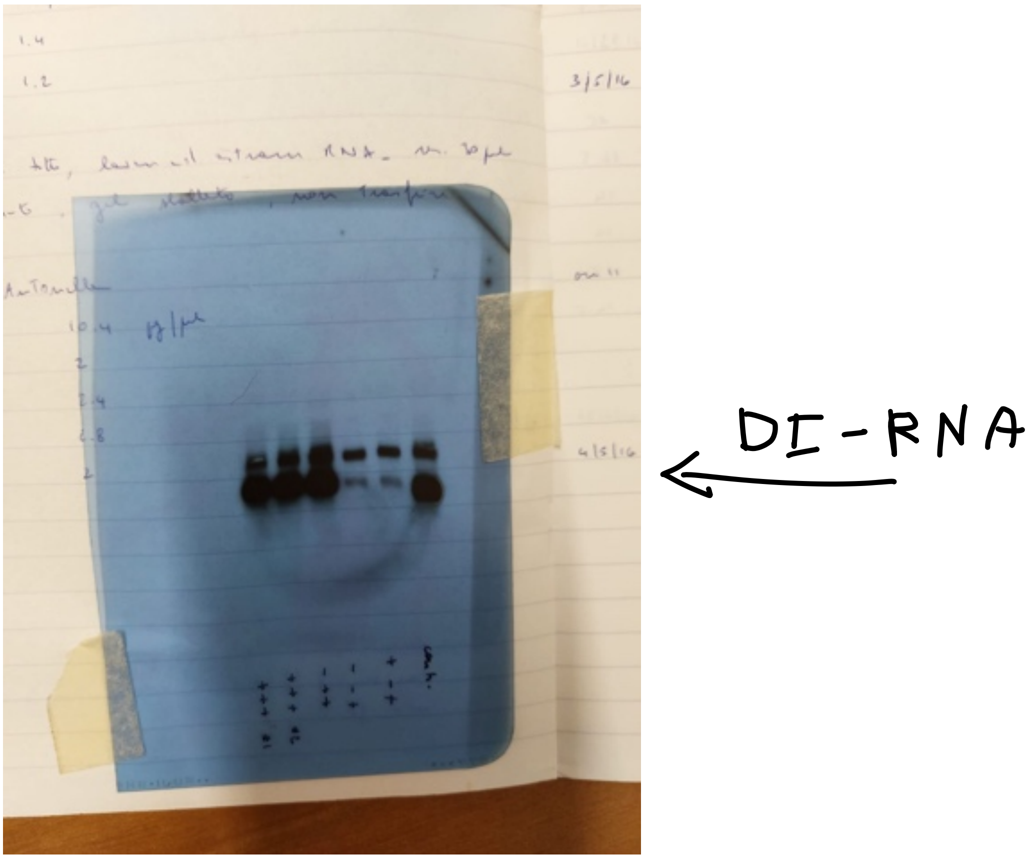

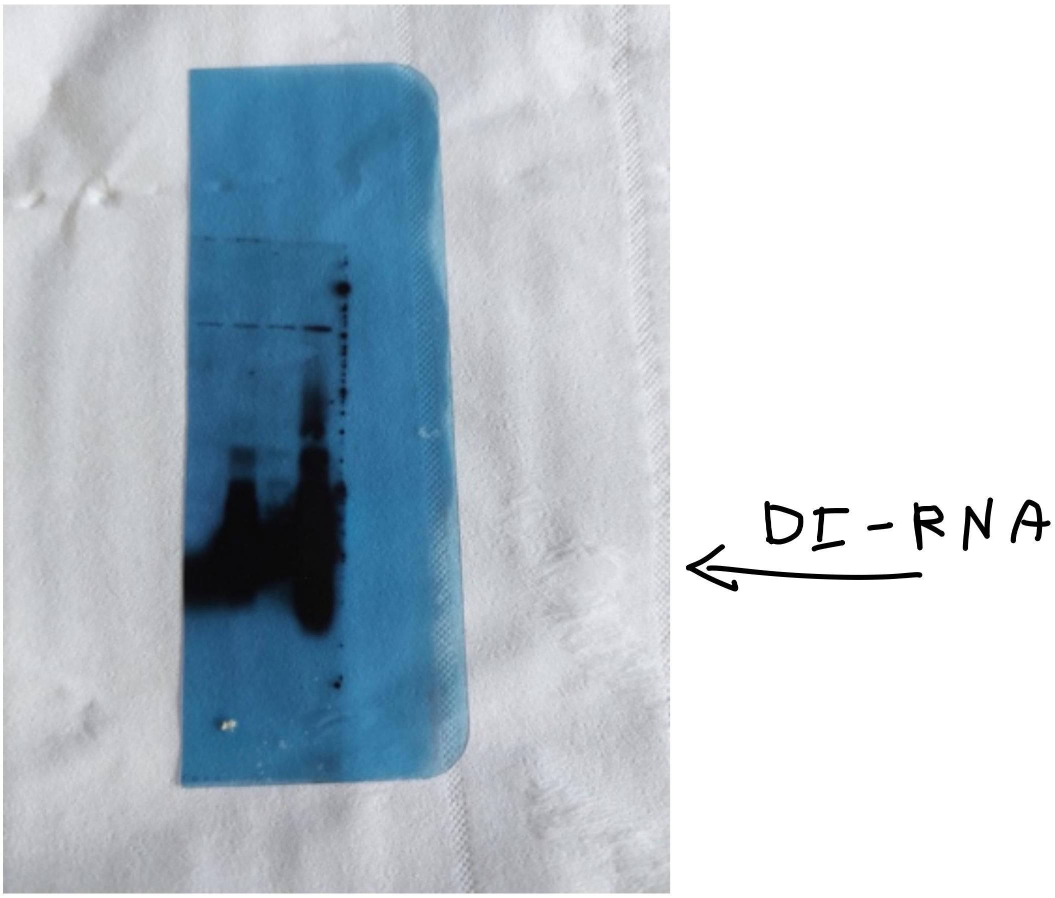

F)

Selected for the main image

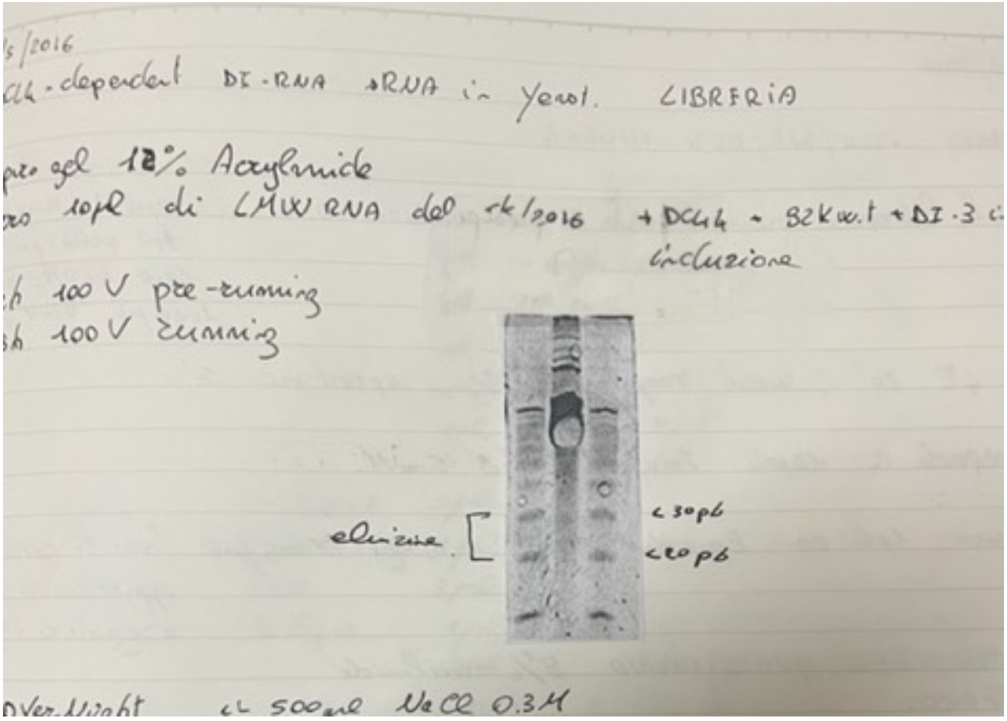

20.04.2018

Procedo con isolamento siRNAs da
e + - + yeast (LUISA)

Gel 12% PAGE denaturante. 1h pre[illegible]
80V

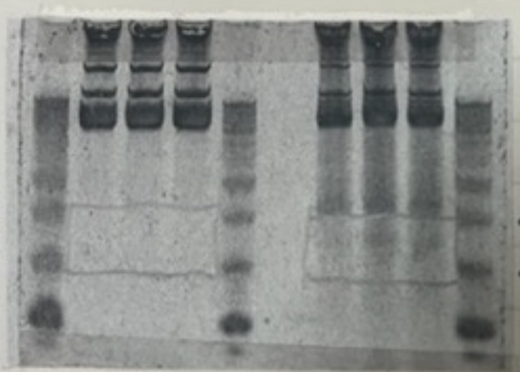

E[illegible]

NaCl 1µl + [illegible]

5/5/2016

Northern blot piccoli RNA arricchiti

Procedo con lavaggio EtOH 70% e pellet dry.
Risospendo in 10µl dd H_2O
Add 10µl RNA loading buffer 70° x 10' ⇒ ICE

Carico gel denaturante 0,5X TBE 12% Acrilamide

pre running 1h,30' 122V

Running 4h 100V
(Bromophenol blue at bottom)

[illegible] staining 5'

Transfer as manual.

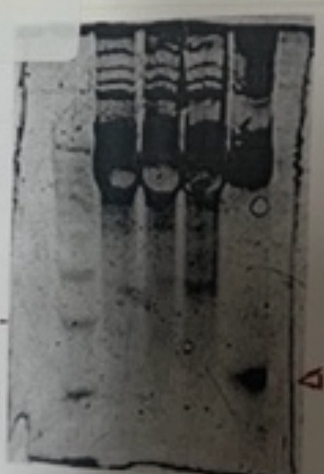

Supplement: Supplementary file 6 — Additional file 6. Replicates Raw Western blots and nucleic acid gels. Replicates Raw Western blots and nucleic acid gels relative to the images at A) Fig. 1B, expression of HADCL4 at different temperatures in S. cerevisiae, B) Fig. 1D, enrichment of HADCL4, C) Fig. 1E, in vitro functionality of yeast crude extract or DEYF in processing Tombusviruses dsRNAs in vitro transcribed, D) Fig. 3B, coexpression of tombusvirus replicase p33 and HADCL4 in yeast, E) Fig. 3C, Northern blot analysis of tombusvirus DI-RNA replication in yeast, F) Fig. 3C Denaturing PAGE of sRNA-enriched fraction stained with EtBr from yeast cells co-expressing HA-DCL4, CymDSV p33/p92 and DI-RNA. [file 11658_2023_469_MOESM6_ESM.pdf]
